# Reproducible lung protective effects of a TGFβR1/ALK5 inhibitor in a bleomycin‐induced and spirometry‐confirmed model of IPF in male mice

**DOI:** 10.14814/phy2.70077

**Published:** 2024-10-11

**Authors:** Asbjørn Graver Petersen, Stefanie H. Korntner, Jamal Bousamaki, Denise Oró, Alba Manresa Arraut, Susanne E. Pors, Casper Gravesen Salinas, Maja Worm Andersen, Martin Rønn Madsen, Yaohui Nie, Jordan Butts, Manuel Roqueta‐Rivera, Ulf Simonsen, Henrik H. Hansen, Michael Feigh

**Affiliations:** ^1^ Gubra Hørsholm Denmark; ^2^ Enanta Pharmaceuticals Watertown Massachusetts USA; ^3^ Department of Biomedicine, Pulmonary and Cardiovascular Pharmacology, Faculty of Health Aarhus University Aarhus Denmark

**Keywords:** ALK5 inhibitor, animal model, bleomycin, deep learning, histopathological scoring, idiopathic pulmonary fibrosis, spirometry, TGFβ receptor, transcriptomics, translatability, whole‐body plethysmography

## Abstract

This study comprehensively validated the bleomycin (BLEO) induced mouse model of IPF for utility in preclinical drug discovery. To this end, the model was rigorously evaluated for reproducible phenotype and TGFβ‐directed treatment outcomes. Lung disease was profiled longitudinally in male C57BL6/JRJ mice receiving a single intratracheal instillation of BLEO (*n* = 10–12 per group). A TGFβR1/ALK5 inhibitor (ALK5i) was profiled in six independent studies in BLEO‐IPF mice, randomized/stratified to treatment according to baseline body weight and non‐invasive whole‐body plethysmography. ALK5i (60 mg/kg/day) or vehicle (*n* = 10–16 per study) was administered orally for 21 days, starting 7 days after intratracheal BLEO installation. BLEO‐IPF mice recapitulated functional, histological and biochemical hallmarks of IPF, including declining expiratory/inspiratory capacity and inflammatory and fibrotic lung injury accompanied by markedly elevated TGFβ levels in bronchoalveolar lavage fluid and lung tissue. Pulmonary transcriptome signatures of inflammation and fibrosis in BLEO‐IPF mice were comparable to reported data in IPF patients. ALK5i promoted reproducible and robust therapeutic outcomes on lung functional, biochemical and histological endpoints in BLEO‐IPF mice. The robust lung fibrotic disease phenotype, along with the consistent and reproducible lung protective effects of ALK5i treatment, makes the spirometry‐confirmed BLEO‐IPF mouse model highly applicable for profiling novel drug candidates for IPF.

## 1 INTRODUCTION

Idiopathic pulmonary fibrosis (IPF) is a chronic interstitial lung disease with unknown etiology. IPF carries a poor prognosis with an estimated survival within 3–5 years upon diagnosis without treatment and represents a high economic burden for individuals and healthcare resources (Ley et al., 2011; Strongman et al., 2018). IPF is clinically manifested as progressive dyspnea, declining lung function and radiologically evident usual interstitial pneumonia and honeycombing reflecting pathological fibrotic remodeling of the lung architecture (Raghu et al., 2018). Although the pathogenesis is incompletely understood, IPF is considered resulting from a combination of genetic and environmental factors (Moss et al., 2022). Prevailing mechanistic hypotheses involve repeated lung microinjuries gradually exhausting alveolar epithelial adaptive and regenerative capacity. At this stage, molecular signals from degenerating epithelial cells can trigger immune cells to recruit and activate resident lung fibroblasts that produce and secrete collagens. Ultimately, persistent fibroblast stimulation will result in excessive fibrotic damage causing irreversible collapse of alveoli and subsequent loss of tissue aeration (Heukels et al., 2019a; Martinez et al., 2017). Despite recent advances in drug development for IPF, lung transplantation remains the only treatment option to restore lung function and improve survival in IPF. Pirfenidone and nintedanib are the only available disease‐modifying pharmacological modalities and these two drugs have been used for many years in the management of IPF (Raghu et al., 2018). Landmark studies have documented that pirfenidone and nintedanib can improve quality of life, attenuate symptoms and slow the rate of lung functional decline, however, progression of IPF is neither halted nor reversed (Canestaro et al., 2016; King et al., 2014; Richeldi et al., 2014). In addition, both agents are associated with adverse gastrointestinal effects that may lead to treatment discontinuation (Corte et al., 2015; Lancaster et al., 2017).

Given the high unmet need for more efficacious antifibrotic therapies in IPF, animal models that can better predict clinical efficacy of drug candidates are highly warranted. Although no single rodent model recapitulates the full spectrum of the human condition, the single‐instillation bleomycin (BLEO)‐induced mouse model of pulmonary fibrosis (BLEO‐IPF) is the best characterized animal model of IPF (Jenkins et al., 2017; Kolb et al., 2020; Tashiro et al., 2017). The BLEO‐IPF mouse is the preferred model in IPF research, however, there several aspects of the BLEO‐IPF mouse which have implications for its applicability in preclinical drug discovery.

Importantly, no standardization of the single‐instillation BLEO‐IPF mouse model exists, which can be challenging when designing, interpreting and comparing study outcomes in the model. Accordingly, several BLEO‐based IPF models are available which differ with respect to mouse strain, BLEO dose/administration route and study duration (Carrington et al., 2018; Tashiro et al., 2017). Also, there is no consensus on critical read‐outs in BLEO‐IPF mouse studies. For example, while forced vital capacity (FVC) is the most employed and accepted endpoint in IPF clinical trials (Nathan & Meyer, 2014), spirometry assessments have only been used to a limited extent in preclinical IPF model studies (Carrington et al., 2018). In the absence of lung functional endpoints, histological and biochemical markers are often applied as surrogate endpoints in BLEO‐induced mice. Although these markers offer a direct measure of fibrosis, changes in lung collagen levels may not necessarily translate to meaningful benefits on lung function. Given that histology and biochemistry is usually performed on discrete lung tissue specimens, it also remains unresolved if local changes in these readouts are representative for the whole lung, which could potentially give rise to misleading study conclusions. Furthermore, histomorphometrics are often complemented by assessment of lung fibrosis severity using the modified clinical Ashcroft scoring system (Ashcroft et al., 1988; Hübner et al., 2008), which is inherently subject to bias due to intra/inter‐observer variability and impractical because of the reliance on resources from expert histopathologists. Therefore, a broad set of well‐validated functional, biochemical and histological methodologies must be applied to achieve robust data in BLEO‐IPF mouse studies.

A key characteristic of the BLEO‐IPF mouse model is the spontaneous regression of pulmonary inflammation and fibrosis after single‐dose BLEO administration (Della Latta et al., 2015; Izbicki et al., 2002; Peng et al., 2013). As a result, the treatment window is limited in the model which makes the timing of drug administration crucial for obtaining conclusive results (Della Latta et al., 2015; Yanagihara et al., 2020). Most pharmacological studies in the model have profiled compounds, including pirfenidone and nintedanib (Schaefer et al., 2011; Wollin et al., 2014), using prophylactic or early interventional dosing regimens (Kolb et al., 2020). While studies aiming to prevent lung fibrosis have contributed to increased knowledge on molecular mechanisms underlying the pathogenesis of IPF, this approach has limited implications as most IPF patients have a significant fibrotic burden upon diagnosis (Ley et al., 2011). Consequently, there is an increasing appreciation that potential IPF drug therapies must be characterized within the relatively stable fibrotic phase in BLEO‐induced mice (Carrington et al., 2018; Jenkins et al., 2017; Tashiro et al., 2017).

Finally, an important barrier to successfully advancing novel treatment concepts is the lack of robustness of preclinical study findings (Drude et al., 2021). For any given animal model, it should therefore be a priority to demonstrate reproducible therapeutic effects of the drug treatment concept in the given animal model (Pound & Ritskes‐Hoitinga, 2018; Tashiro et al., 2017). Although it has been recommended to implement rigorous study designs and replication protocols when evaluating drug candidates in the BLEO‐IPF mouse (Jenkins et al., 2017), it remains to specifically pharmacologically validate the BLEO‐IPF mouse model for reproducible drug therapeutic outcomes.

Collectively, it is essential to establish a standardized BLEO‐IPF mouse model framework in preclinical drug discovery. We, therefore, aimed to rigorously evaluate the single‐installation BLEO‐IPF mouse model for reproducible lung disease phenotype and TGFβ‐targeted drug therapeutic outcomes using a wide array of state‐of‐the‐art lung functional, biochemical and histological methods.

## 2 MATERIALS AND METHODS

### 2.1 Animals

Male C57BL/6JRj mice were purchased from Janvier Labs (Le Genest Saint Isle, France) and housed in a controlled environment (12 h light/dark cycle, 21 ± 2°C, humidity 50 ± 10%). Animals had ad libitum access to tap water and chow (Altromin 1324; Brogaarden, Hoersholm, Denmark). Each animal was identified by an implantable subcutaneous microchip (PetID Microchip, E‐vet, Haderslev, Denmark). Body weight and clinical signs (lack of grooming, inactivity, cold to the touch, piloerection, squinted eyes, weight loss, and respiratory distress) were assessed daily during the study. Humane endpoints were based on veterinary clinical evaluation and included ≥20% body weight loss within 5 days, and/or respiratory distress exceeding moderate stress load.

### 2.2 Disease phenotyping studies in BLEO‐IPF mice

12–14‐week‐old mice received a single intratracheal instillation of BLEO sulphate (Baxter Healthcare, Deerfield, IL; 1.5 mg/kg, dissolved in 50 μL sterile saline; BLEO‐IPF mice). Untreated mice served as healthy controls (CTRL). Body weight was measured once daily. Only animals that showed a mild to moderate body weight loss (≥−5% and ≤ −17%) at day 6 post‐administration were randomized into study groups. Animals were terminated on days 7 (*n* = 10), 14 (*n* = 12), 21 (*n* = 12), 28 (*n* = 12), 35 (*n* = 12), and 42 (*n* = 7), respectively, after BLEO administration. Whole‐body plethysmography (WBP, see below) was performed in a separate cohort of CTRL (*n* = 10) and BLEO‐IPF mice (*n* = 11) on days 7, 14, 21, and 28 after BLEO administration, followed by terminal spirometry on day 28. Progressive development of pulmonary fibrosis was associated with an overall mortality rate of 20.7% in BLEO‐IPF mice as determined over a period of 7–42 days after a single intratracheal instillation of BLEO. All animals were euthanized according to the humane endpoints described above.

### 2.3 ALK5i treatment studies in BLEO‐IPF mice

TGFβ1R/ALK5 inhibitor (ALK5i) treatment was characterized in a total of 6 independent intervention studies in BLEO‐IPF mice. Animals received a single intratracheal instillation of BLEO (2.0 mg/kg, dissolved in 50 μL sterile saline) or saline (CTRL). BLEO‐IPF mice were randomized and stratified to treatment according to baseline pulmonary function (PenH 0.7–2.0, primary factor) assessed by WBP, and body weight (secondary factor) measured on day 7 after BLEO administration. ALK5i (60 mg/kg/day, cat. No. #406415, MedKoo Biosciences; Durham, NC) or vehicle was administered orally by gavage (5 mL/kg) for 21 days. ALK5i was dissolved in equimolar of 1 M HCl, and 0.5 % methylcellulose in Milli‐Q water was added until the final concentration was reached. ALK5i was profiled in two different dosing regimens (three studies per regimen), that is, bi‐daily (BID) dosing of vehicle (study 1, *n* = 14; study 2, *n* = 14; study 3, *n* = 14) or ALK5i (30 mg/kg, dosing at 6:30 am and 2:30 pm; study 1, *n* = 10; study 2, *n* = 16; study 3, *n* = 10), or once daily (QD) dosing of vehicle (study 4, *n* = 14; study 5, *n* = 10; study 6, *n* = 11) or ALK5i (60 mg/kg, dosing at 6:30 am; study 4, *n* = 15; study 5, *n* = 12; study 6, *n* = 14), respectively. Mice orally administered vehicle (BID or QD) served as controls (CTRL, *n* = 10 per study). Body weight was measured once daily. BLEO‐IPF mice showed an overall mortality rate of 18.8% in the ALK5i studies. All animals were euthanized according to the humane endpoint described above.

### 2.4 Unrestrained Whole‐body plethysmography

To evaluate non‐invasive lung functional parameters before allocation to treatment intervention, respiration was measured using a whole‐body plethysmograph (vivoFlow, EMKA Technologies, Paris, France). Each animal was placed in an individual plethysmography chamber supplied with air and was allowed to move freely. Rate and volume of respiration of unrestrained mice were recorded and ventilatory parameters were averaged over a period of 15 min. See Table S1 for a description of the individual WBP variables.

### 2.5 Pulmonary spirometry

After terminal blood sampling (see below), animals were sacrificed by cervical dislocation followed by tracheostomy. An 18G, 10 mm metal cannula was placed in the trachea and a tight fit was secured with a suture. Animals were then connected to the flexiVent system (SCIREQ, Canada). To standardize lung volume and calculate inspiratory capacity, a deep inflation was performed by inflating the mouse lungs to a pressure of 30 cm H_2_O over a period of 3 s and then held at that pressure for another 3 s. Pressure‐volume (PV) loops were generated to obtain static compliance of the respiratory system. The negative pressure‐driven forced expiratory maneuver was performed by inflating the mouse lungs to a pressure of 30 cmH_2_O over 1 s, hold this pressure for 2 s before connecting the animal's airways to the negative pressure reservoir (−50 cm H_2_O) for 2 s. Forced expired volume measured over 0.1 s (FEV0.1) and FVC were calculated directly from the flow‐volume loops generated during lung deflation. All maneuvers were performed in triplicate. A coefficient of determination of 0.95 was set as the lower limit for accepting a measurement. See Table S1 for a description of the individual spirometry variables.

### 2.6 Blood and tissue sampling

Blood samples were kept on ice and centrifuged (10 min, 4°C, 3000 g) to generate EDTA‐stabilized plasma. The supernatant was aliquoted and stored at −70°C for further analysis. The lung was rinsed with 3 × 0.5 mL of cold sterile saline to collect bronchoalveolar lavage fluid (BALF). BALF was kept on ice, and centrifuged (7 min, 4°C, 400 ×g); the supernatant was aliquoted and stored at −70°C for further analysis. The lungs were excised and weighed. Thereafter, the right lung lobe was isolated by cutting the right bronchi and stored at −70°C until processing for biochemistry (superior and middle lobe) and RNA sequencing (inferior lobe), respectively. The left lung was used for histology. To reduce the number of mice used in the study, random tissue sampling was not applied.

### 2.7 Lung biochemistry

The right superior lobe was homogenized in 6 M HCl and hydrolyzed to degrade collagen, centrifuged and hydroxyproline (HP) content was measured in the supernatant using a colorimetric kit (Quickzyme Biosciences, Leiden, The Netherlands). A separate study was conducted to compare whole‐lobule HP content in the right inferior, right superior and left lung lobule following BLEO administration. Plasma and BALF surfactant protein D (SPD) was measured with the mouse SPD quantikine ELISA Kit (#MSFPD0, R&D systems, Minneapolis, MN). All three TGFβ isoforms in plasma, BALF and lung tissue were quantified by the mouse U‐PLEX TGFβ Combo MSD Kit (#K1542K, Meso Scale Discovery, Rockville, MD). For analysis of SMAD3 and p38MAPK phosphorylation, lung tissues were lysed in RIPA buffer with protease/phosphatase inhibitors (#78446, Thermo Fisher Scientific, Waltham, MA). Protein concentration was determined by BCA colorimetric assay (#23225, Thermo Fisher Scientific). Equal amount of 50 μg total protein was used for ELISA‐based quantification of phospho‐SMAD3 (pS423/pS425, #85‐86,192‐11, Thermo Fisher Scientific) and phospho‐p38MAPK (pT180/pY182, #85‐86,022‐11, Thermo Fisher Scientific).

### 2.8 Lung histology

The left lung lobe was cannulated through the trachea and perfusion‐fixed with 10% neutral‐buffered formalin (NBF) at a constant fluid pressure for 5 min. The left lung was transferred to a vial containing 10% NBF and immersion‐fixed overnight at room temperature, transferred to 70% ethanol and stored at 4°C until further processing. The tissue was then placed in a Histokinette to infiltrate prior to embedding. The lung was sectioned at 4 μm thickness on a microtome and mounted on Starfrost slides (Knittel, Braunschweig, Germany). Sections were stained with Masson's trichrome (MT, Sigma‐Aldrich, Brøndby, Denmark), Picro Sirius red (PSR, Sigma‐Aldrich, Brøndby, Denmark), anti‐type I collagen (Col1a1, cat. 1310‐01; Southern Biotech, Birmingham, AL), anti‐type III collagen (Col3, cat. 1330‐01, Southern Biotech, Birmingham, AL), anti‐alpha‐smooth muscle action (α‐SMA, cat. Ab124964; Abcam, Cambridge, UK), or anti‐galectin‐3 (Gal‐3, cat. 125,402, Biolegend, San Diego, CA) using standard procedures. Slides were scanned using a 20× objective (Aperio AT2, Leica Biosystems). Deep learning‐based image analysis was applied for automated histopathological scoring using the Ashcroft scoring system (see below). Quantitative histomorphometry was performed using digital imaging software (Visiomorph; Visiopharm, Hørsholm, Denmark) for the determination of whole‐section lung fibrosis (PSR, Col1a1, Col3), fibroblast activation (α‐SMA) and inflammation (Gal‐3), respectively, expressed as positive staining relative (%) to the total sectional area.

### 2.9 Ashcroft scoring using automated deep learning‐based image analysis

AI‐assisted pathology (Gubra Histopathological Objective Scoring Technology [GHOST]) was developed in Python 3.7 to grade lung fibrosis using the scoring system established by Ashcroft et al. (Ashcroft et al., 1988; Hübner et al., 2008). MT‐stained and scanned lung tissue sections were 50% downscaled and split into tiles of 512 × 512 pixels, whereafter each tile was assigned a score based on a convolutional neural network (CNN) model trained to reproduce the Ashcroft grading system. A total 93 lung sections and 4666 tiles were manually scored according to the Ashcroft criteria (grade 0–8; Table S2) by an expert histopathologist blind to experimental groups. An additional class was used for tiles showing non‐alveolar tissue, such as large bronchi or blood vessels, which were therefore excluded from the scoring. Before training, data was resampled to balance the classes and divided into training (75%), validation (20%), and test (5%) sets. The CNN trained model was used to compute the Ashcroft score in lung samples. The CNN model was trained based on the Inception‐v3 network architecture (Szegedy et al., 2016) using the Keras library (Chollet et al., 2015) to predict the tile score. The training was performed for 25 epochs, and the accuracy was computed at every iteration. The Adam optimizer (Kingma & Ba, 2015) was used during training, and data augmentation was applied in the form of rotations, flips, and brightness. The trained CNN model showed a Cohens kappa value of 0.78 measured on a test set of 269 samples. A composite Ashcroft score, expressed as whole‐section score, was calculated as mean score of all tiles in the individual lung section. The final Ashcroft score for a given lung sample was computed as the mean score of all tiles calculated using the following formula:

$$\begin{array}{c} \left(0\times \text{n0}\right)+\left(1\times \text{n1}\right)+\left(2\times \text{n2}\right)+\left(3\times \text{n3}\right)+\left(4\;x\;\text{n4}\right)\\ +\left(5\times \text{n5}\right)+\left(6\times \text{n6}\right)+\left(7\times \text{n7}\right)+\left(8\times n8\right)\\ /\text{n0}+\text{n1}+\text{n2}+\text{n3}+\text{n4}+\text{n5}+\text{n6}+\text{n7}+\text{n8} \end{array}$$

where nx was the number of tiles in each grade.

### 2.10 Lung stereology

A separate study in BLEO‐IPF mice was conducted to evaluate whole‐lobule distribution of BLEO‐induced fibrotic injury. Mice received a single intratracheal instillation of BLEO sulphate (2.0 mg/kg, dissolved in 50 μL sterile saline, BLEO‐IPF mice, *n* = 11) or saline (CTRL mice, *n* = 8). Perfusion‐fixed left lung tissues were fixed in 10% NBF for 5 min, and then kept in 10% NBF overnight. The volume of the left lung was obtained using the Archimedes principle. The weight of the fluid displaced by the immersed left lung was measured as described previously (Ochs & Schipke, 2021). After paraffin‐embedding, the left lung was sectioned at 4 μm thickness on a microtome and mounted on Superfrost Plus slides. A total of 6–8 sections per lung, with a distance of 300 μm between sections, were obtained for quantitative Col1a1 immunohistochemistry. Col1a1 staining was expressed as proportionate (%) area of whole‐section area for a representative single‐section, average %‐area for all serial sections sampled, and whole‐lobule Col1a1 volume (% area of Col1a1 × total lobule volume, μm^3^) using digital image analysis (Visiomorph; Visiopharm, Hørsholm, Denmark).

### 2.11 Lung RNA sequencing

The inferior lobe was dissected and stored at −70°C for RNA sequencing analysis. Samples were homogenized in lysis buffer and RNA was purified using NucleoSpin® RNA binding strips (cat. no. 740698.5, Macherey‐Nagel, Dueren, Germany). RNA purity and concentration were measured using a NanoDrop 2000 and cDNA libraries were prepared using NEBNext® Ultra II Directional RNA library prep Kit for Illumina® (cat. no. E7760L, New England Biolabs, Ipswich, MA). cDNA libraries were sequenced to a depth of approximately 15 million reads per sample (single‐end, 75 bp reads) on a NextSeq 500 System (Illumina, San Diego, CA) using the NextSeq 500/550 High Output Kit version 2.5 (cat. no. 20024906, Illumina). Reads were aligned to the GRCm38 release 96 Ensembl *Mus musculus* genome using STAR version 2.7.0f (Dobin et al., 2013). All downstream analyses were performed with R version 3.6.0 (R Core Team, 2018). For differential gene expression analysis, the R package DESeq2 (Love et al., 2014) was used and p‐values were corrected for multiple testing using the Benjamini‐Hochberg method (5% False Discovery Rate, FDR <0.05). Lung global gene expression changes in BLEO‐IPF mice were validated against previously reported lung RNA transcriptome changes in patients with advanced IPF undergoing lung transplantation (*n* = 36) compared to human controls donor lungs deemed ineligible for lung transplantation (*n* = 17) (Sivakumar et al., 2019). In addition, a curated set of 179 gene expression markers linked to human IPF and lung fibrosis (Ma et al., 2022; McDonough et al., 2019; Roach et al., 2021; Wollin et al., 2015; Zhao et al., 2022) were probed in BLEO‐IPF mice. Gene set enrichment analysis was conducted using the R package Piano (Väremo et al., 2013).

### 2.12 Flow cytometric analysis of bronchoalveolar lavage fluid

After centrifugation of the BALF, the cell pellet was resuspended in 250 μL of cell staining buffer (PBS 2% FBS 5 mM EDTA) and strained through a 60 μm filter to obtain a single‐cell suspension. Samples were blocked with anti‐CD16/CD32 antibody TruStain fcX™ (Biolegend, San Diego, CA), incubated with the viability marker Zombie Aqua™ (BioLegend, San Diego, CA) and subsequently stained with one of two antibody panels to phenotype lymphoid cells [CD45 PE‐Cy7 (clone I3/2.3), CD11b BV650 (clone M1/70), CD3 FITC (clone KT3.1.1), CD4 BV421 (clone GK1.5), CD8 APC (clone 53‐6.7), NK1.1 PE (clone PK136), B220 BV605 (clone RA3‐6B2), and CD19 APC Fire 750 (clone 6D5) and myeloid cells [CD45 PE‐Cy7 (clone I3/2.3), CD11b BV650 (clone M1/70), Ly6G BV605 (clone 1A8), Ly6C APC (clone HK1.4), F4/80 BV421 (clone T45‐2342) and CD11c (clone N418)], and subsequently fixed. Prior to analysis, cells were passed through a 60 μm filter, 50 μL of CountBright™ counting beads (Invitrogen, Carlsbad, CA) were added to each sample and flow cytometry was performed on a 4‐laser CytoFlex S (Beckman Coulter, Indianapolis, IN). Data was analyzed using the Cy13) tExpert 2.2 software (Beckman Coulter, Indianapolis, IN). Viable CD45^+^ leucocytes without debris and doublets were displayed on a CD45/CD11b dot plot and gated for CD45^+^CD11b^+^ myeloid cells and CD45^+^CD11b‐ lymphoid cells. Neutrophils were segmented from viable CD45^+^ leucocytes as Ly6G^+^Ly6C^+^ population. Non‐neutrophils (Ly6G^–^Ly6C^–^) were displayed on a F4/80/CD11c dot plot. Interstitial macrophages and alveolar macrophages were classified as F4/80^+^CD11c^low^ and F4/80^+^CD11c^hi^ cells, respectively (Bedoret et al., 2009; Dipiazza et al., 2017; Misharin et al., 2013; Yu et al., 2016; Zaynagetdinov et al., 2013). Alveolar macrophages were further divided into CD11b^+^ and CD11b^–^ subpopulations. Dendritic cells were selected as F4/80^–^CD11c^+^ cells. Lymphoid cells were divided into a CD3^+^ and CD3^–^ fraction. In the CD3^+^ fraction, based on co‐expression of CD4 and CD8 antigens, three subpopulations of T lymphocytes were established: T‐helper cells (CD4^+^CD8^–^), cytotoxic T‐cells (CD4^–^CD8^+^), and double negative (DN) T‐cells (CD4^–^CD8^–^). B cells were selected from the CD3^–^ fraction as CD19^+^ and B220^+^ cells.

### 2.13 Statistics

Except from deep learning‐based image analysis and RNA sequencing, data were analyzed using GraphPad Prism version 10.1.2 software (GraphPad, La Jolla, CA). All results are shown as mean ± standard error of mean (S.E.M.). After confirmation of normal distribution of the data (D'Agostino and Pearson omnibus normality test), a one‐way analysis of variance (ANOVA) followed by Dunnett's multiple comparisons test was applied. When the above assumption was violated, non‐parametric statistics and Kruskal‐Wallis test was utilized, followed by Dunn's multiple comparisons test. A *p* value ≤0.05 was considered statistically significant.

## 3 RESULTS

### 3.1 Biomarkers of lung disease in BLEO‐IPF mice

**FIGURE 1:**
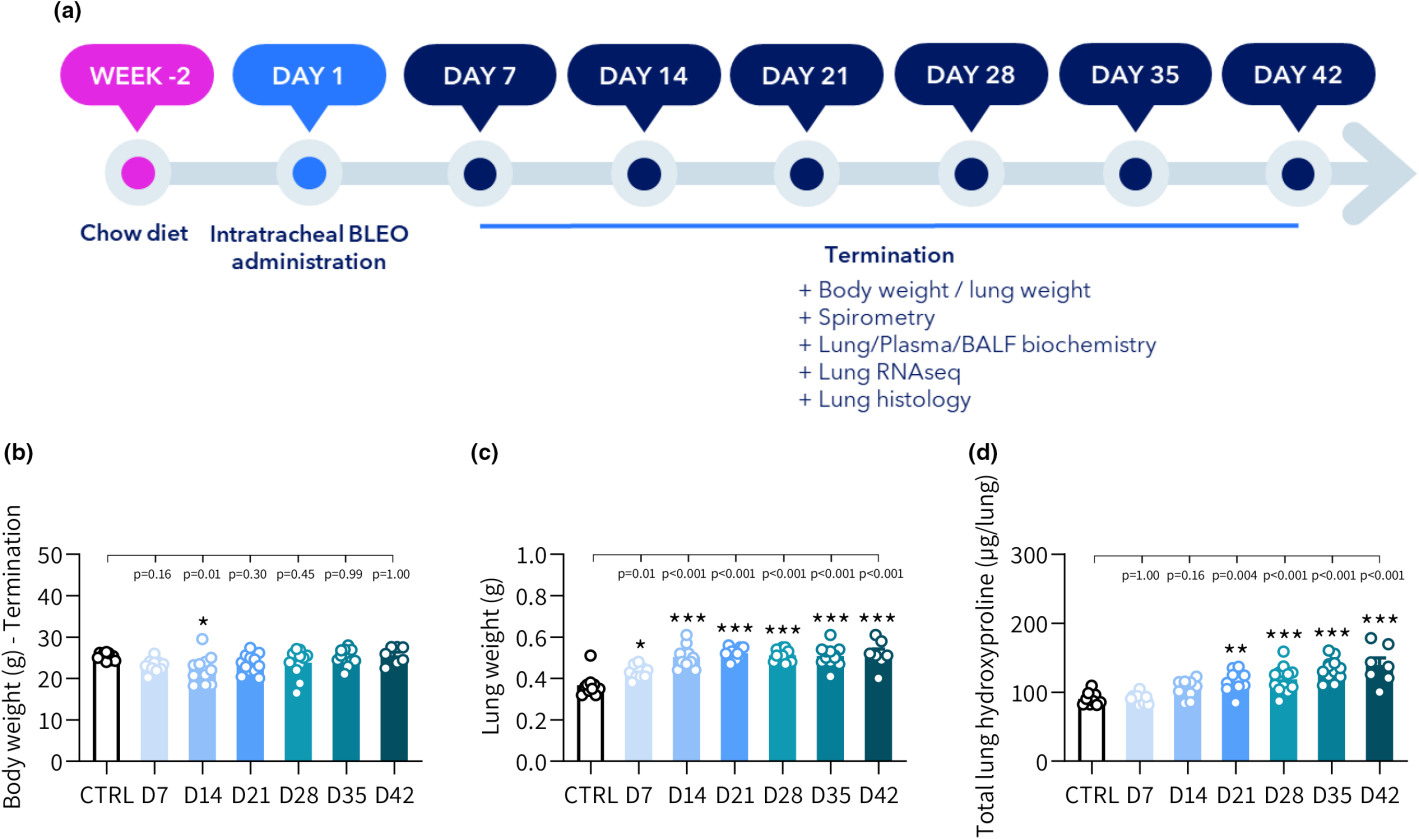
Longitudinal study in BLEO‐IPF mice. (a) Study outline. Mice received a single intratracheal instillation of saline vehicle (*n* = 10) or bleomycin (BLEO‐IPF, 1.5 mg/kg) and were terminated on day 7‐42 after BLEO administration (*n* = 7–12 per time point). (b) Terminal body weight. (c) Lung weight. (d) Total lung hydroxyproline (HP) content. *p < 0.05, **p < 0.01, ***p < 0.001 vs. CTRL. Dunnett's test one‐factor linear model.

Markers of lung injury were profiled longitudinally in BLEO‐IPF mice and compared to untreated controls (CTRL), see study outline in Figure 1a. While body weight was relatively stable over time in BLEO‐IPF mice (Figure 1b), lung weight significantly increased (reflecting oedema and inflammation) and reached a plateau from day 14 after BLEO administration (Figure 1c). Lung HP levels increased progressively over the entire study, attaining statistical significance from day 21 and onwards (Figure 1d). Temporal and tissue‐specific dynamics were detected for SPD and TGFβ biomarkers in BLEO‐IPF mice (Table 1). Compared to CTRL, plasma SP‐D levels peaked on day 14 followed by a gradual decline. In contrast, BALF SP‐D levels remained significantly elevated in BLEO‐IPF mice during the entire study. In CTRL mice, TGFβ levels were as follows: TGFβ1 > TGFβ2> > TGFβ3 (plasma), TGFβ2 > TGFβ1 > TGFβ3 (BALF), and TGFβ1> > TGFβ2> > TGFβ3 (lung tissue). Compared to CTRL mice, BLEO‐IPF mice showed unchanged plasma TGFβ1 and TGFβ2 levels but lower TGFβ3 concentrations. All TGFβ isoforms were significantly elevated in BALF samples from BLEO‐IPF mice, showing most pronounced increases on day 14–21. Lung levels of TGFβ2 and TGFβ3, but not TGFβ1, were significantly increased in BLEO‐IPF mice from day 14 and onwards.

**TABLE 1:** BLEO‐IPF mice show increased levels of surfactant protein D (SPD) and TGFβ biomarkers.

	CTRL *n* = 10	BLEO‐IPF D7 *n* = 10	CTRL vs. D7	BLEO‐IPF D14 *n* = 11	CTRL vs. D14	BLEO‐IPF D21 *n* = 12	CTRL vs. D21	BLEO‐IPF D28 *n* = 12	CTRL vs. D28	BLEO‐IPF D35 *n* = 12	CTRL vs. D35	BLEO‐IPF D42 *n* = 6	CTRL vs. D42
Plasma SP‐D (ng/ml)	5.4 ± 0.4	793 ± 104	*p* = 0.13	1661 ± 378***	*p* <0.001	929 ± 173*	*p* = 0.04	1031 ± 378*	*p* = 0.02	311 ± 127	*p* = 0.87	14.8 ± 5.6	*p* >0.99
Plasma TGFβ1 (pg/ml)	9023 ± 530	12,730 ± 3140	*p* = 0.37	8774 ± 309	*p* >0.99	9093 ± 940	*p* >0.99	11,150 ± 2102	*p* = 0.81	11,934 ± 1069	*p* = 0.56	3895 ± 1405**	*p* = 0.004
Plasma TGFβ2 (pg/ml)	2356 ± 66	2155 ± 79	*p* = 0.22	2253 ± 40	*p* = 0.77	2418 ± 57	*p* = 0.95	2470 ± 110	*p* = 0.70	2582 ± 65	*p* = 0.11	*ND*	*N/A*
Plasma TGFβ3 (pg/ml)	17 ± 1.9	5.2 ± 1.3**	*p* = 0.001	7.2 ± 1.4**	*p* = 0.007	7.2 ± 2.0**	*p* = 0.006	15 ± 3.8	*p* = 0.98	4.9 ± 1.5**	*p* = 0.003	3.7 ± 0.7**	*p* = 0.005
BALF SP‐D (ng/ml)	517 ± 39	2690 ± 410**	*p* < 0.001	1950 ± 248***	*p* < 0.001	2370 ± 236***	*p* < 0.001	2220 ± 270***	*p* < 0.001	2750 ± 220***	*p* < 0.001	642 ± 81	*p* = 0.23
BALF TGFβ1 (pg/ml)	19 ± 3.1	75 ± 11*	*p* = 0.04	115 ± 13***	*p* < 0.001	132 ± 16***	*p* < 0.001	91 ± 14**	*p* = 0.005	83 ± 15*	*p* = 0.01	*ND*	*N/A*
BALF TGFβ2 (pg/ml)	61 ± 3.6	468 ± 74***	*p* < 0.001	676 ± 84***	*p* < 0.001	611 ± 85***	*p* < 0.001	382 ± 68**	*p* = 0.006	289 ± 48	*p* = 0.09	42 ± 9.4	*p* = 0.07
BALF TGFβ3 (pg/ml)	8.0 ± 0.3	10 ± 0.5**	*p* = 0.001	12 ± 0.3***	*p* < 0.001	11 ± 0.4***	*p* < 0.001	8.0 ± 0.5	*p* > 0.99	7.6 ± 0.4	*p* = 0.86	2.1 ± 0.4***	p < 0.001
Lung TGFβ1 (pg/mg)	1088 ± 127	1065 ± 91	*p* > 0.99	940 ± 75	*p* = 0.64	928 ± 103	*p* = 0.55	690 ± 52**	*p* = 0.008	829 ± 62	*p* = 0.14	*ND*	*N/A*
Lung TGFβ2 (pg/mg)	99 ± 4.5	126 ± 6.8	*p* = 0.46	192 ± 15***	*p* < 0.001	166 ± 15**	*p* = 0.001	206 ± 15***	*p* < 0.001	194 ± 9.6***	*p* < 0.001	165 ± 8.7***	*p* < 0.001
Lung TGFβ3 (pg/mg)	6.9 ± 0.2	8.8 ± 0.5	*p* = 0.96	20 ± 1.4***	*p* < 0.001	18 ± 2.5**	*p* = 0.002	25 ± 3.5***	*p* < 0.001	19 ± 2.1**	*p* = 0.001	7.2 ± 1.0	*p* = 0.78

*Note*: Mice received a single intratracheal instillation of bleomycin (BLEO‐IPF, 1.5 mg/kg) and were terminated on days 7, 14, 21, 28, 35 and 42, respectively, after BLEO administration (*n* = 6–12 per group). Mice receiving an intratracheal dose of saline served as healthy controls (*n* = 10). Mean ± S.E.M. *p < 0.05, **p < 0.01, ***p < 0.001 vs. CTRL, one‐way ANOVA with Dunnett's multiple comparisons test or Kruskal‐Wallis test with Dunn's multiple comparisons test.

Abbreviations: BALF, bronchoalveolar lavage fluid; NA, not applicable; ND, not determined; TGFβ1‐3, transforming growth factor‐β isoforms 1‐3.

### 3.2 Combined spirometry and WBP confirms impaired pulmonary function in BLEO‐IPF mice

**FIGURE 2:**
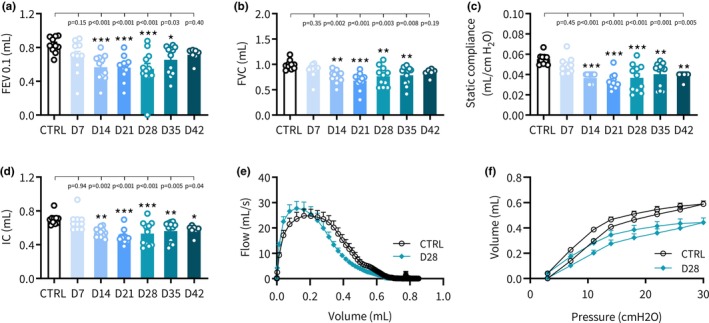
Progressive dynamics in pulmonary function in BLEO‐IPF mice. Respiratory physiology assessment using spirometry. (a) Forced expiratory volume in 0.1 seconds (FEV0.1). (b) Forced vital capacity (FVC). (c) Static compliance. (d) Inspiratory capacity (IC). (e) Flow‐volume curves for healthy controls (CTRL) and BLEO‐IPF mice (28 days after BLEO instillation). (f) Pressure‐volume curves for healthy controls (intratracheal instillation of saline vehicle, CTRL) and BLEO‐IPF mice (28 days after BLEO instillation). *p < 0.05, **p < 0.01, ***p < 0.001 vs. CTRL. Dunnett's test one‐factor linear model.

**FIGURE 3:**
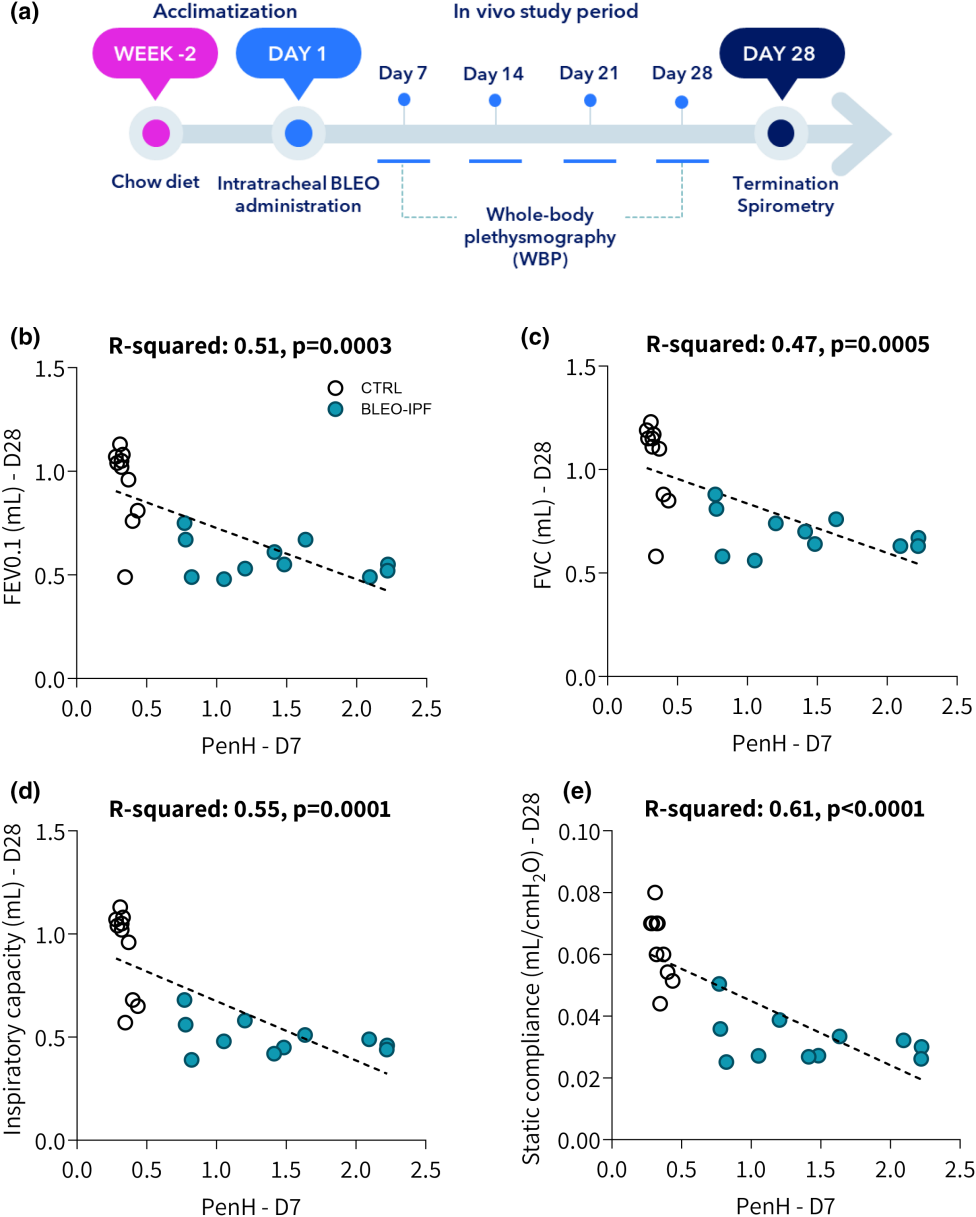
Whole‐body plethysmography (WBP) in freely moving BLEO‐IPF mice. (a) Outline of the characterization study in BLEO‐IPF mice using repeated WBP assessment of respiratory function in individual mice on days 7, 14, 21, and 28 after bleomycin (BLEO) induction (see Figure S1 for all WBP data). Mice received a single intratracheal instillation of saline vehicle (CTRL, *n* = 10) or bleomycin (BLEO‐IPF, 2.0 mg/kg, *n* = 11). Terminal spirometry was performed on day 28. Correlation of baseline WBP (PenH, day 7) and terminal spirometry endpoints (day 28), including (b) forced expiratory volume in 0.1 seconds (FEV0.1), (c) forced vital capacity (FVC), (d) inspiratory capacity (IC), and (e) static compliance, respectively. Simple linear regression analysis.

BLEO‐IPF mice demonstrated impaired lung function, indicated by significantly lowered expiratory function (FEV0.1, FEV), static compliance and inspiratory capacity, see Figure 2a–d. Impaired lung function was manifest from day 14‐28 whereupon all four spirometry endpoints gradually declined towards normal control levels. While expiratory function (FEV0.1, FEV) was not significantly different from controls on day 42, static compliance and inspiratory capacity remained significantly reduced. Flow‐volume and PV curves obtained on day 28 further supported lung functional deficits in BLEO‐IPF mice compared to controls (Figure 2e,f). In contrast to terminal spirometry, WBP is a non‐invasive procedure whereby respiratory function/capacity can be assessed in unrestrained conscious animals, making WBP ideal for confirming respiratory deficits (Hoymann, 2007; Lomask, 2006). An additional intervention study was therefore performed in BLEO‐IPF mice to monitor lung deficits by WBP on day 7 (baseline) as well as day 14, 21 and 28 after BLEO administration followed by terminal spirometry at day 28 (Figure 3a). WBP parameters progressively changed over the course of the study (Figure S1). Enhanced pause (PenH), a dimensionless index related to ventilatory timing, was determined as the most robust WBP endpoint in BLEO‐IPF mice. PenH peaked on day 7 and remained significantly elevated over the course of the study as compared to healthy controls. BLEO‐IPF mice also demonstrated a highly consistent decline in all terminal spirometry variables (Figures S1 and S2). Baseline WBP (PenH) was inversely correlated to all terminal spirometry parameters compared to healthy controls (*p* < 0.001, Figure 3b–e). By confirming good correlation between baseline WBP (PenH) and body weight vs. terminal gold‐standard spirometry, baseline WBP (PenH) and body weight was applied to randomize and stratify disease severity in BLEO‐IPF mice before treatment (see below).

### 3.3 Automated deep learning‐based Ashcroft scoring of lung fibrosis in BLEO‐IPF mice

**FIGURE 4:**
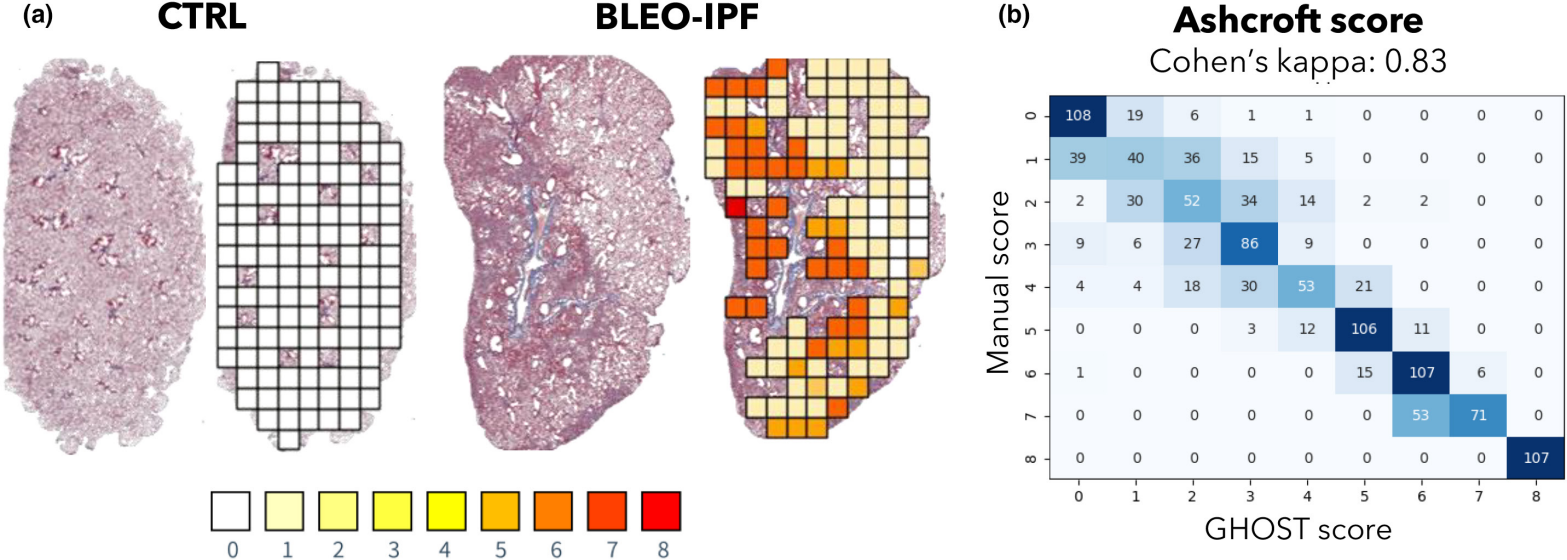
Validation of automated deep learning‐based Ashcroft scoring of lung fibrosis in BLEO‐IPF mice. (a) Deep learning‐based Ashcroft scoring, using Gubra Histopathological Objective Scoring Technology (GHOST), applied to the entire left lung at 10x magnification. Representative Masson's trichome stainings used for image analysis. Heatmaps depict Ashcroft scores (score 0‐8; i.e., normal lung tissue architecture to total fibrous obliteration) in individual lung image tiles of 512 × 512 pixels. (b) Ashcroft score was computed and validated using a test set of lung samples from a total of 93 mice. There was a high concordance between manual and automated (GHOST) scoring (kappa value of 0.83).

An automated AI‐based digital image analysis pipeline (GHOST) was developed to obtain a more accurate and objective method for scoring of lung fibrosis as outlined by Ashcroft et al. (Ashcroft et al., 1988; Hübner et al., 2008). The Ashcroft score was computed and validated using a test set of MT‐stained lung tissue samples from 93 individual mice (Figure 4a). The analysis indicated a high degree of agreement between automated and manual Ashcroft scoring (Kappa value of 0.83; Figure 4b). This establishes GHOST as an accurate AI‐based method for unbiased and automated Ashcroft scoring of pulmonary fibrosis in BLEO‐IPF mice.

### 3.4 Histological disease progression in BLEO‐IPF mice

**FIGURE 5:**
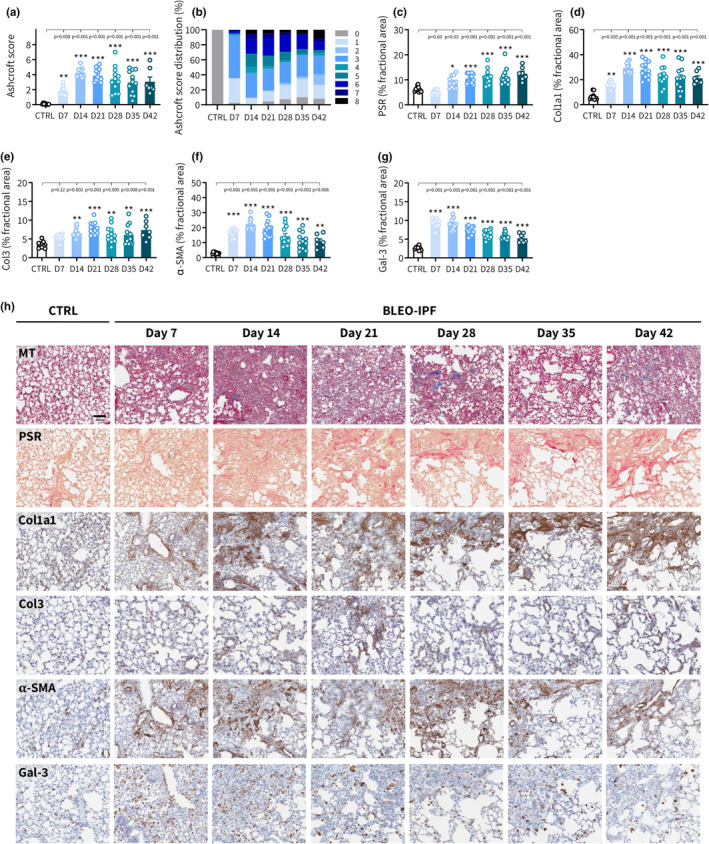
Histological hallmarks of fibrotic lung disease in BLEO‐IPF mice. (a) GHOST‐based Ashcroft scoring on day 7‐42 after BLEO administration (*n* = 7–12 per group). Histopathological scoring was performed on sections stained with Masson's trichrome (MT). **p < 0.01, ***p < 0.001 vs. healthy controls (intratracheal instillation of saline vehicle, CTRL, *n* = 10); Dunnett's test one‐factor linear model. (b) Group‐wise distribution of Ashcroft scores. (c–g) Histomorphometric assessment of fibrosis (PSR, Col1a1, Col3), fibrogenesis (α‐SMA) and inflammation (Gal‐3) using conventional image analysis. Data were calculated as proportionate (%) area of staining. (c) PSR. (d) Collagen‐1a1 (Col1a1); (e) Collagen‐3 (Col3). (f) α‐SMA. (g) Galectin‐3 (Gal‐3). *p < 0.05, **p < 0.01, ***p < 0.001 vs. CTRL). Dunnett's test one‐factor linear model. (h) Representative photomicrographs. Scale bar, 100 μm.

GHOST was subsequently implemented for objective and reproducible assessment of Ashcroft scores in BLEO‐IPF mice. Whereas CTRL mice displayed normal lung tissue, BLEO‐IPF mice demonstrated incremental increase in fibrosis score from day 7 (mean score 1.8 ± 0.2) to day 14 (mean score 4.3 ± 0.2), whereafter fibrosis severity slightly declined over the remainder of the study (day 42, mean score 3.1 ± 0.6), see Figure 5a. The temporal dynamics in fibrosis severity was reflected by corresponding changes in the within‐group distribution of Ashcroft scores (Figure 5b).

Quantitative histology was performed to further evaluate lung disease progression in BLEO‐IPF mice. As histomorphometrics was applied to a subset of sections from the left lobule, we initially validated BLEO‐induced changes in histological markers for being representative of whole‐lobe changes. To this end, we applied stereological analysis on Col1a1‐stained lung tissue sections sampled systematically throughout the left lung lobule of BLEO‐IPF and saline control mice, respectively (Figure S3a,b). Controls and BLEO‐IPF mice demonstrated low within‐group variation in Col1a1 staining at all lobule levels evaluated. For each lobule level, BLEO‐IPF mice showed a consistently higher %‐area of Col1a1 as compared to controls (Figure S3c). Single‐section Col1a1 levels were representative for mean whole‐lobule levels (Figure S3d). Stereological volume analysis further supported robust increases in whole‐lobule Col1a1 expression in BLEO‐IPF mice (Figure S3e). In addition, changes in biochemical (HP) and histological markers (Ashcroft score, PSR, Col1a1, Col3, α‐SMA, Gal‐3) were similar across all lung lobules (left lung lobule, right inferior lobule, right superior lobule) in BLEO‐IPF mice (Figure S4), indicating that changes in left lung lobule markers of fibrosis and inflammation were representative for whole‐lung changes in the model.

Histomorphometric analyses confirmed dynamic changes in lung fibrosis and inflammation in BLEO‐IPF mice. While PSR levels progressively increased from day 14 and onwards (Figure 5c), Col1a levels peaked on day 14 and thereafter gradually declined but remained significantly increased over the entire monitoring period (Figure 5d). Col3 levels were also significantly increased, reaching a plateau from day 14–21 (Figure 5e). Expression of α‐SMA, a marker of fibrogenesis, peaked on day 14 followed by a gradual decline (Figure 5f). Gal‐3 immunostaining indicated significant pulmonary inflammation on day 7–14 with a consistent regression profile from day 21 (Figure 5g). Representative histological images are shown in Figure 5h. Pulmonary inflammation was supported by flow cytometry analysis of BALF samples (measured on day 21). BLEO‐IPF mice showed significant expansions in both myeloid and lymphoid immune cell populations (Figure S5a–d). Enrichment in myeloid cells was primarily driven by increases in alveolar and interstitial macrophage subsets as well as dendritic cells and neutrophils (Figure S5e–J). Substantial increases in T‐cells (CD4^+^CD8^–^ T‐helper cells, CD4^–^CD8^+^ cytotoxic T‐cells, CD4^–^CD8^–^ T‐cells) and B‐cells were also detected in BLEO‐IPF mice (Figure S5k–o).

### 3.5 Clinical translatability of lung transcriptome changes in BLEO‐IPF mice

**FIGURE 6:**
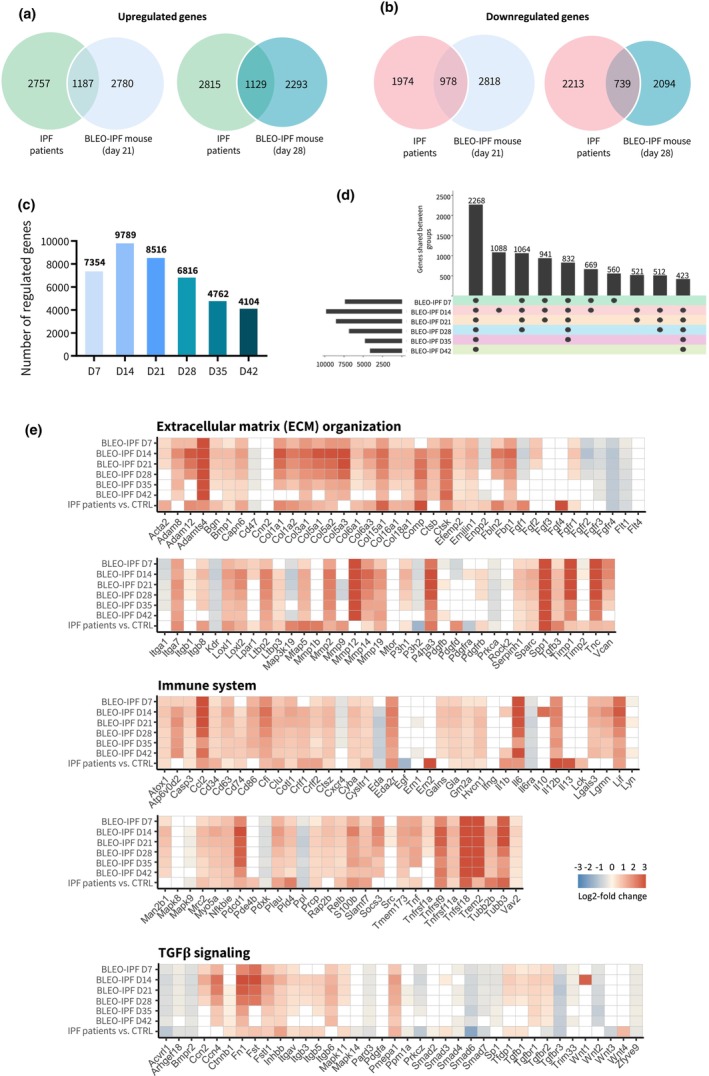
Progressive lung transcriptome changes in BLEO‐IPF mice validated against lung RNA sequencing data from IPF patients. (a, b) Venn diagrams depicting shared and separate differentially expressed genes (DEGs; false discovery rate <0.05) in BLEO IPF mice vs. patients with advanced IPF (Sivakumar et al., 2019). BLEO‐IPF mice (day 21 and 28 post‐administration (*n* = 12 per group) compared to intratracheal saline administration (*n* = 10). (c) Total number of DEGs in BLEO‐IPF mice (day 7‐42 post‐BLEO administration) compared to saline controls. (d) Number of DEGs shared between BLEO‐IPF mice at different time points after BLEO administration. (e) Curated list of 179 candidate genes linked to IPF pathogenesis and fibrosis, divided into three categories, that is, extracellular matrix (ECM) organization, immune system and TGFβ‐associated signaling. Color gradients indicate significantly upregulated (red color) or downregulated (blue color) genes compared to corresponding controls. White color indicates no significant change in gene expression compared to corresponding controls.

Global lung gene expression changes in BLEO‐IPF mice were compared to lung transcriptome signatures reported in patients with advanced IPF (Sivakumar et al., 2019). Venn diagrams illustrate the degree of overlap between significantly upregulated and downregulated genes detected in BLEO‐IPF mice (day 21 and 28) and IPF patients as compared to corresponding controls (Figure 6a,b). An extensive number of differentially expressed genes (DEGs) were determined in lung samples from BLEO‐IPF mice, with alterations being most pronounced on day 14 (*n* = 9789 DEGs; Figure 6c). A relatively large proportion of DEGs (*n* = 2268) remained significantly regulated over the entire study period in BLEO‐IPF mice (Figure 6d). To obtain further resolution of lung transcriptome regulations, RNA sequencing data were probed for candidate genes linked to human IPF pathology and fibrosis (Ma et al., 2022; McDonough et al., 2019; Roach et al., 2021; Sivakumar et al., 2019; Wollin et al., 2015; Zhao et al., 2022). A total of 179 curated candidate genes were segmented into three major functional categories (extracellular matrix (ECM) organization, immune system, TGFβ signaling). BLEO‐IPF mice and IPF patients showed similar directional and significant shifts in candidate gene expression (Figure 6e). Compared to controls, BLEO‐IPF mice demonstrated significant and widespread gene regulations, involving several pathways associated with ECM organization, the immune system and TGF‐β signaling, being generally manifest from day 7 after BLEO administration (Figure 6e, Figure S6,S7). While a wide range of markers of immune cell showed sustained upregulation, several tended to gradually lower their expression towards normalization. Dynamic shifts in ECM remodeling were also observed during the course of lung disease BLEO‐IPF mice. For example, expression of *Acta2*, *Col1a1*, *Col1a2*, *Col5a1*, *Col6a1*, *Col6a3* and *Col16a1* were gradually normalized while *Col3a1*, *Col5a2*, *Col5a3*, *Col15a* and *Col18a1* remained at high levels. Changes in the expression of TGFβ‐associated molecular signaling markers were apparent in BLEO‐IPF mice (Figure 6e; Figure S7). Accordingly, a subset of canonical TGFβR1 (also known as activin receptor‐like kinase 5, Alk5 (Derynck & Budi, 2019; Massagué, 2012)) signaling transduction markers were upregulated (*Tgfb1*, *Tgfbr1*) while others were unaffected (*Smad2*, *Smad3*). Other TGFβ‐responsive genes implicated in IPF, of which some are molecular targets for approved or clinical‐stage IPF therapeutics (Ma et al., 2022; Roach et al., 2021; Wollin et al., 2015; Zhao et al., 2022), were consistently upregulated (*e.g., Ccn2* (*Ctgf*), *Ccn4* (*Wisp1*), *Fn1, Fst, Inhbb, Itgav, Itgb5, Itgb6, Mapk11, Pmepa1, Tgfbr2*), downregulated (e.g., *Acvrl1, Prkcz, Smad6, Tgfbr3, Wnt2*), or largely unregulated (e.g., *Mapk14, Pdgfa, Smad7, Sp1, Wnt3*) over the course of the study.

### 3.6 Reproducible therapeutic outcomes of ALK5i treatment in BLEO‐IPF mice

**FIGURE 7:**
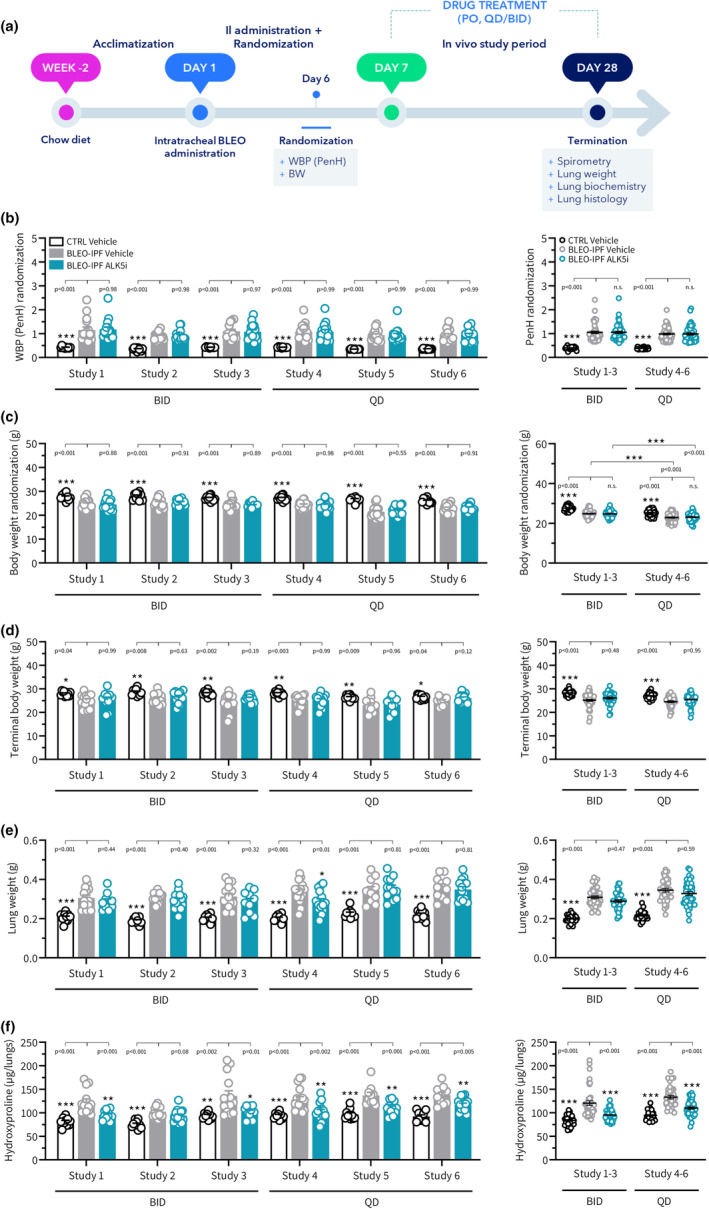
ALK5i treatment effects on body weight, lung weight and lung hydroxyproline content in BLEO‐IPF mice. TGFβR1/ALK5 inhibitor (SB525334) treatment outcomes were compared in 6 independent intervention studies in BLEO‐IPF mice. (a) Study outline. Mice received a single intratracheal instillation of saline vehicle (CTRL) or bleomycin (BLEO‐IPF, 2.0 mg/kg). BLEO‐IPF mice received vehicle (*n* = 10‐14 per study) or ALK5i (60 mg/kg/day, *n* = 10–16 per study), administered orally as bi‐daily (30 mg/kg, BID; study 1‐3) or once daily (60 mg/kg, QD; study 4–6) dosing for 21 days, starting 7 days after intratracheal BLEO instillation. Vehicle‐dosed CTRL mice served as healthy controls (*n* = 10 per study). BLEO‐IPF mice were randomized and stratified to treatment according to (b) PenH (primary factor, determined by whole‐body plethysmography, WBP) and (c) baseline body weight (secondary factor), measured on day 6 after bleomycin administration. (d) Terminal body weight. (e) Lung weight. (f) Total lung hydroxyproline (HP) content. *Left panels*: Data in individual mice according to group and study. *p < 0.05, **p < 0.01, ***p < 0.001 vs. BLEO‐IPF Vehicle, one‐way ANOVA with Dunnett's test for multiple comparisons. *Right panels*: Composite analysis of Alk5i treatment outcomes (group average). ***p < 0.001 vs. corresponding groups in study 1‐3, one‐way ANOVA with Dunnett's test for multiple comparisons.

For intervention studies, a slightly higher intratracheal dose of BLEO (2.0 mg/kg) was applied to promote a further robust and homogenous lung disease phenotype while preserving a relatively low mortality rate. Further increments in the BLEO dose (2.5 mg/kg) were not tolerated (data not shown). To characterize TGFβ‐directed therapy in the BLEO‐IPF mouse, we profiled a standard orally active ALK5i [SB525334, (Grygielko et al., 2005)] in the model. An initial study aimed to assess drug‐target engagement after ALK5i treatment for 21 days (30 mg/kg, BID), starting at 7 days after BLEO administration. Lung tissue levels of phospho‐SMAD3, a marker of canonical TGFβR1/ALK5 receptor signaling, were significantly upregulated in vehicle‐dosed BLEO‐IPF mice and this effect was completely reversed by ALK5i (Figure S8a). In contrast, phospho‐p38MAPK, a marker of non‐canonical TGFβR1/ALK5 receptor signaling, was unchanged in BLEO‐IPF mice and not influenced by ALK5i (Figure S8b), suggesting preferential recruitment of canonical TGFβR1/ALK5 receptor signaling upon ALK5i treatment. In addition, a subsequent lung global gene expression analysis indicated that several TGF‐β responsive genes were significantly downregulated by ALK5i, including *Ccn2* (*Ctgf*), *Ccn4* (*Wisp1*), *Itgav*, *Itgb5*, *Itgb6, Pdgfa, Pmepa1* and *Tgfbr1* (Figure S8c). Following confirmation of pulmonary target engagement of ALK5i, the compound was subsequently profiled in an extensive series of intervention studies in BLEO‐IPF mice to evaluate consistency of ALK5i treatment outcomes on functional, biochemical, and histological endpoints. ALK5i was administered either bi‐daily (30 mg/kg, BID; 3 studies) or once daily (60 mg/kg, QD; 3 studies) for 21 days. Except for comparing two routes of ALK5i administration, study designs were identical in all six studies in BLEO‐IPF mice (Figure 7a). In all studies, BLEO‐IPF mice were randomized and stratified to treatment according to WBP (PenH) and baseline body weight measured on day 7 after BLEO administration (Figure 7b,c).

**FIGURE 8:**
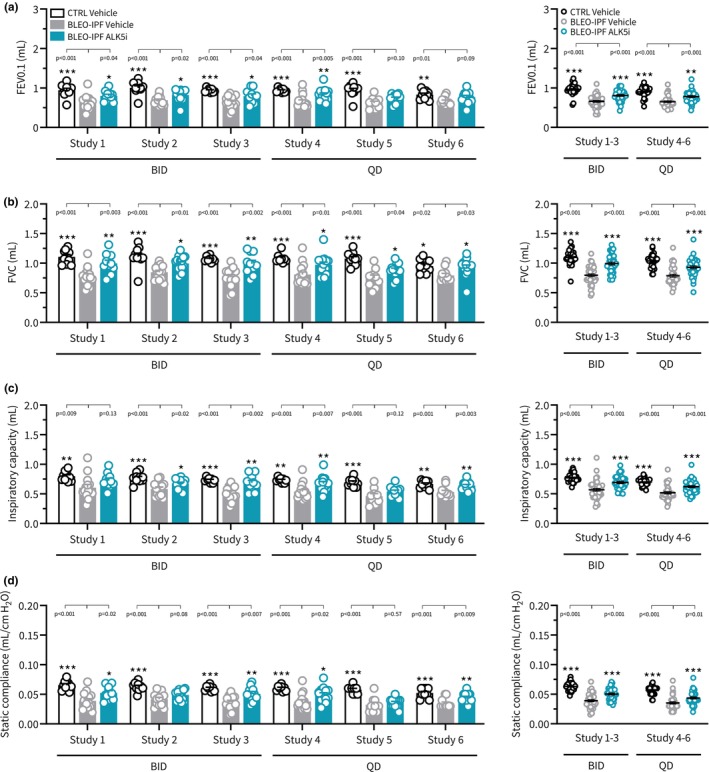
ALK5i treatment effects on spirometry endpoints in BLEO‐IPF mice. Outcomes of TGFβR1/ALK5 inhibitor (SB525334) treatment were compared in 6 independent intervention studies performed in BLEO‐IPF mice. Mice received a single intratracheal instillation of saline vehicle (CTRL) or bleomycin (BLEO‐IPF, 2.0 mg/kg). Mice received vehicle (*n* = 10‐14 per study) or ALK5i (60 mg/kg/day, *n* = 10‐16 per study), administered orally as bi‐daily (30 mg/kg, BID; study 1‐3) or once daily (60 mg/kg, QD; study 4‐6) dosing for 21 days, starting 7 days after intratracheal BLEO instillation. Vehicle‐dosed CTRL mice served as healthy controls. (a) Forced expired volume over 0.1 seconds (FEV0.1). (b) Forced vital capacity (FVC). (c) Inspiratory capacity. (d) Static compliance. *Left panels*: Data in individual mice according to group and study. *p < 0.05, **p < 0.01, ***p < 0.001 vs. corresponding BLEO‐IPF Vehicle group, one‐way ANOVA with Dunnett's test for multiple comparisons. *Right panels*: Composite analysis of Alk5i treatment outcomes (group average). **p < 0.01, ***p < 0.001 vs. BLEO‐IPF Vehicle, one‐way ANOVA with Dunnett's test for multiple comparisons.

**FIGURE 9:**
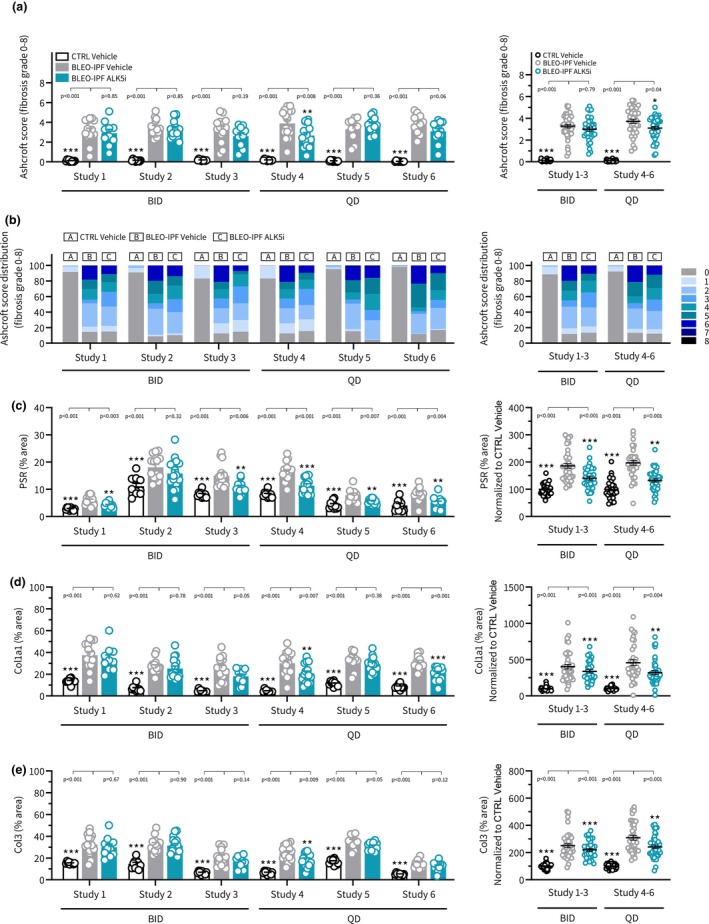
ALK5i treatment effects on lung fibrosis histological endpoints in BLEO‐IPF mice. Outcomes of TGFβR1/ALK5 inhibitor (SB525334) treatment were compared in six independent intervention studies performed in BLEO‐IPF mice. Mice received a single intratracheal instillation of saline vehicle (CTRL) or bleomycin (BLEO‐IPF, 2.0 mg/kg). Mice received vehicle (*n* = 10–14 per study) or ALK5i (60 mg/kg/day, *n* = 10–16 per study), administered orally as bi‐daily (30 mg/kg, BID; study 1–3) or once daily (60 mg/kg, QD; study 4–6) dosing for 21 days, starting 7 days after intratracheal BLEO instillation. Vehicle‐dosed CTRL mice served as healthy controls. (a, b) GHOST‐based Ashcroft fibrosis scoring. (c–e) Histomorphometric assessment of fibrosis (PSR, Col1a1, Col3) using conventional image analysis. Data were calculated as proportionate (%) area of histological staining. (c) PSR. (d) Collagen‐1a1 (Col1a1). (e) Collagen‐3 (Col3). *Left panels*: Data in individual mice according to group and study. **p < 0.01, ***p < 0.001 vs. vs. corresponding BLEO‐IPF Vehicle group. One‐way ANOVA with Dunnett's test for multiple comparisons. *Right panels*: Composite analysis of Alk5i treatment outcomes (group average), depicted as change vs. corresponding CTRL Vehicle group (for quantitative histology only). *p < 0.05, **p < 0.01, ***p < 0.001 corresponding BLEO‐IPF Vehicle group, one‐way ANOVA with Dunnett's test for multiple comparisons.

**FIGURE 10:**
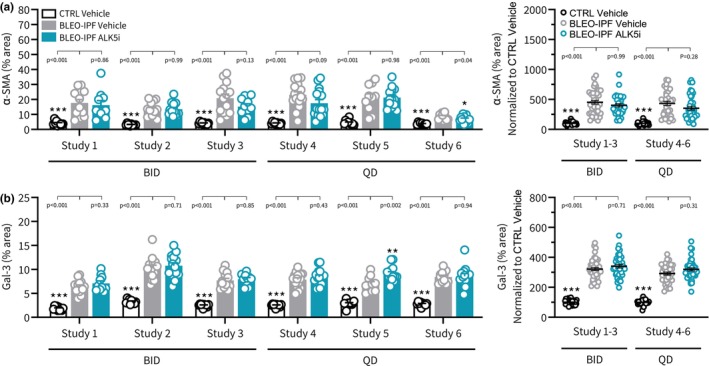
ALK5i Treatment effects on lung fibrogenesis and inflammation histological endpoints in BLEO‐IPF mice. Outcomes of TGFβR1/ALK5 inhibitor (SB525334) treatment were compared in six independent intervention studies performed in BLEO‐IPF mice. Mice received a single intratracheal instillation of saline vehicle (CTRL) or bleomycin (BLEO‐IPF, 2.0 mg/kg). Mice received vehicle (*n* = 10‐14 per study) or ALK5i (60 mg/kg/day, *n* = 10–16 per study), administered orally as bi‐daily (30 mg/kg, BID; study 1–3) or once daily (60 mg/kg, QD; study 4–6) dosing for 21 days, starting 7 days after intratracheal BLEO instillation. Vehicle‐dosed CTRL mice served as healthy controls. Histomorphometric assessment of fibrogenesis (α‐SMA) and inflammation (Gal‐3) using conventional image analysis. Data were calculated as proportionate (%) area of histological staining. (a) Alpha‐smooth muscle actin (α‐SMA). (b) Galectin‐3 (Gal‐3). *Left panels*: Data in individual mice according to group and study. *p < 0.05, **p < 0.01, ***p < 0.001 vs. corresponding BLEO‐IPF Vehicle group. One‐way ANOVA with Dunnett's test for multiple comparisons. *Right panels*: Composite analysis of Alk5i treatment outcomes (group average), depicted as change vs. corresponding CTRL Vehicle group. ***p < 0.001 vs. corresponding BLEO‐IPF Vehicle group, one‐way ANOVA with Dunnett's test for multiple comparisons.

**FIGURE 11:**
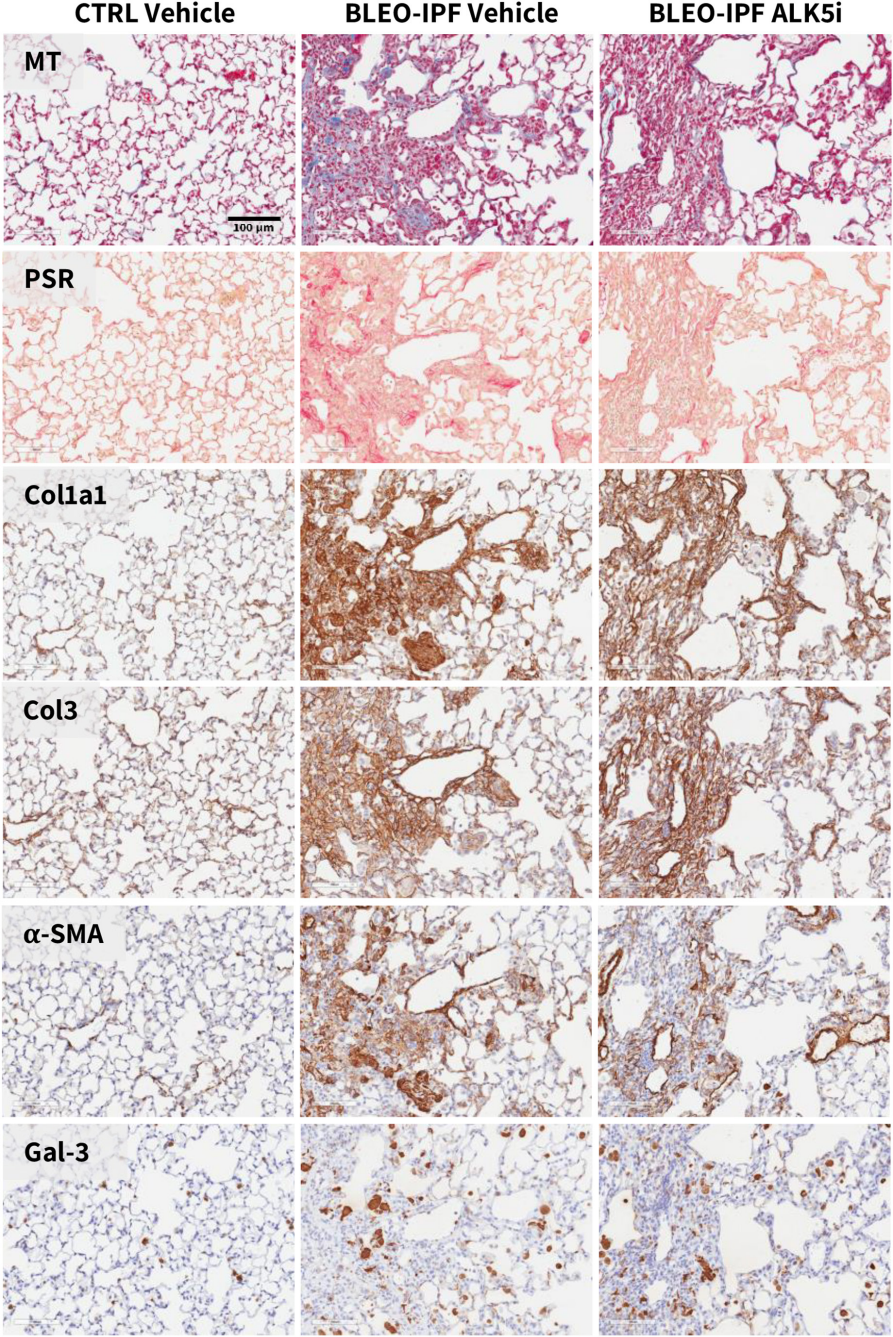
Representative photomicrographs of lung histological stainings. Histological staining from representative ALK5i treatment study (QD dosing) in BLEO‐IPF mice. Mice receiving a single intratracheal instillation of saline vehicle served as healthy controls (CTRL Vehicle). α‐SMA, α‐smooth muscle actin; Col1a1, collagen‐1a1; Col3, collagen‐3; Gal‐3, galectin‐3; MT, Masson's trichrome; PSR, picro sirius red. Scale bar, 100 μm.

Irrespective of regimen (BID or QD), vehicle‐dosed BLEO‐IPF mice showed similar disease phenotype at termination across all six studies. Compared to healthy control mice, vehicle‐dosed BLEO‐IPF mice showed moderate (≈10%) body loss at termination (Figure 7d) with significantly increased lung weight and total lung HP content (Figure 7e,f). BLEO‐IPF control mice demonstrated consistently impaired respiratory outcomes, as reflected by impaired FEV0.1, FVC, inspiratory capacity and static compliance (Figure 8a–d). In agreement with reduced static compliance, a proxy for tissue elasticity, all six studies revealed robust and significant increases in the total set of histological markers of fibrosis (Ashcroft score, PSR, Col1a1, Col3), fibrogenesis (α‐SMA) and inflammation (Gal‐3), see Figures 9, 10, and 11.

The two ALK5i dosing regimens tested (BID and QD) promoted similar and reproducible therapeutic benefits on several pulmonary endpoints in the series of BLEO‐IPF mouse intervention studies performed. Overall, similar therapeutic benefits of ALK5i treatment intervention were achieved for BID and QD dosing regimens, respectively. ALK5i was weight‐neutral and had no consistent effect on lung weight in BLEO‐IPF mice (Figure 7d,e). ALK5i significantly reduced total lung HP content in 5 out of 6 studies (Figure 7f). Notably, ALK5i consistently improved lung function in BLEO‐IPF mice, including expiratory function (FEV0.1, 4 out of 6 studies; FVC, 6 out of 6 studies, Figure 8a,b), inspiratory capacity (4 out of 6 studies, Figure 8c) and static compliance (4 out of 6 studies, Figure 8d). While Ashcroft score was only significantly reduced in 1 out of 6 studies (Figures 9a,b and 11), %‐area of PSR, a standard quantitative histological marker of fibrosis, was significantly decreased in 5 out of 6 studies (Figures 9c and 11). In comparison, ALK5i had less consistent therapeutic effect on other histological markers of fibrosis/fibrogenesis, including Col1a1 (2 out of 6 studies, Figures 9d and 11), Col3 (1 out of 6 studies, Figures 9e and 11) α‐SMA (1 out of 6 studies, Figures 10a and 11). ALK5i did not suppress Gal‐3 expression (Figures 10b and 11).

## 4 DISCUSSION

We here provide a comprehensive description of functional, biochemical, histopathological, and molecular changes over the course of lung disease in the BLEO‐induced and spirometry‐confirmed mouse model of IPF. Our data indicate a consistent disease phenotype in BLEO‐IPF mice with an optimal time window for characterizing fibrosis intervention therapies from days 7–28 after a single intratracheal BLEO instillation. Also, changes in molecular markers of pulmonary fibrosis and immune system dysregulation in the BLEO‐IPF mouse model are comparable with those reported in IPF patients. Importantly, the current study for the first time provides clear evidence of highly reproducible lung functional, biochemical, and histological therapeutic effects of a TGFβR1/ALK5 inhibitor, based on outcomes of six individual intervention studies, establishing ALK5i as a reliable reference drug in preclinical drug discovery for IPF.

The BLEO mouse model is extensively used for studying molecular mechanisms of pulmonary fibrosis and serves as the most widely used animal model in preclinical drug discovery for IPF (Jenkins et al., 2017). A common feature of BLEO‐induced mouse models is the lack of chronicity, as pulmonary fibrosis is not progressive and gradually resolves with time (Degryse & Lawson, 2011; Della Latta et al., 2015; Tashiro et al., 2017). Most BLEO mouse studies have profiled aspects of lung injury at single time points, using a wide range of dosing regimens with single dose intratracheal instillation being the most frequent route of administration (Srour & Thébaud, 2015; Williamson et al., 2015). Although few longitudinal studies on lung pathology have been reported in BLEO mice, they differ based on BLEO dose, route of administration, endpoints applied and duration of disease monitoring (Chung et al., 2003; Milton et al., 2012; Peng et al., 2013; Polosukhin et al., 2012; Washimkar et al., 2023). Given the lack of consistency across laboratories, this study aimed to further inform about temporal dynamics in the disease phenotype and qualify the single BLEO instillation model for use in preclinical drug discovery.

Initially, we characterized functional, biochemical, and histological hallmarks of lung injury in BLEO‐IPF mice for up to 42 days after single‐dose intratracheal administration of BLEO. Overall, our longitudinal study emphasizes the presence of three distinct phases of lung disease in BLEO‐IPF mice, involving an early inflammatory phase (≤7 days), an intermediate phase of active/sustained fibrosis (7–28 days), and a late remodeling phase characterized by gradual resolution of fibrosis (≥35 days). The timing and transition from an acute lung inflammatory response to the onset, progression and regression of fibrosis is in overall agreement with previously reported studies in BLEO‐IPF mice (Izbicki et al., 2002; Peng et al., 2013). Consistent with acute inflammation, a substantial number of immune regulatory genes were found most markedly upregulated from day 7–14 after BLEO administration in our model. Early inflammatory events are a crucial driver of fibrosis in IPF (Ogawa et al., 2021; Seyran et al., 2023; Shi et al., 2021). Pulmonary inflammation in BLEO‐IPF mice was primarily reflected by expansion in alveolar and interstitial macrophage populations as well as T and B lymphocyte subsets. Alveolar macrophages are key players in the initiation and resolution of inflammation (Pound & Ritskes‐Hoitinga, 2018), a major source of TGF‐β (Szegedy et al., 2016), and capable of exacerbating pulmonary fibrosis by stimulating fibroblast proliferation and collagen synthesis (Chollet et al., 2015; Kingma & Ba, 2015). While less investigated, interstitial macrophages can present antigen and acquire a pro‐fibrotic phenotype depending on stimuli (Aran et al., 2019; Meziani et al., 2018). Although the role of the adaptive immune system in IPF pathogenesis remains to be fully elucidated, several T and B cell populations have been implicated in the initiation and perpetuation of pulmonary injury and fibrosis (Heukels et al., 2019b; Spagnolo et al., 2022). In the present study, a role of macrophages in BLEO‐induced inflammation was supported by upregulated Gal‐3 expression which gradually wore off after peaking on day 7. Gal‐3 is highly expressed and secreted by macrophages (Zaynagetdinov et al., 2013), stimulated by TGF‐β receptor signaling and recognized as a pro‐fibrotic factor in IPF (MacKinnon et al., 2012; Mathur & Singh, 2023).

BLEO‐IPF mice consistently developed restrictive respiratory function associated with reduced lung elasticity as determined by spirometry at study termination. While expiratory function was transiently impaired from day 14–28, inspiratory capacity and static compliance remained compromised throughout the monitoring period. Sustained deficits in static compliance, a proxy for tissue elasticity, were consistent with progressive increases in lung HP content and PSR staining. To further assess histological features of fibrosis in BLEO‐IPF mice, we applied Ashcroft's scoring system originally developed to grade fibrosis in human lung specimens (Moss et al., 2022; Raghu et al., 2018), and subsequently validated and adopted for use in rodent models (Ashcroft et al., 1988; Hübner et al., 2008). As for any single ordinal manual scoring system, the Ashcroft scoring is subject to significant intra/interscorer variability (Ashcroft et al., 1988; Hübner et al., 2008). To circumvent these limitations, we developed, validated, and implemented an automated deep learning‐based digital imaging analysis pipeline, termed GHOST, for more accurate and objective Ashcroft scoring of fibrosis on MT‐stained lung sections from BLEO‐IPF mice. As opposed to maintained high levels of PSR staining, Ashcroft scores declined after an initial increase from day 7‐14, signifying gradual normalization of pulmonary collagen architecture. While PSR and MT are instrumental to visualize and quantify collagen fibers, it is not possible to distinguish between collagen fiber types with these colorimetric methods. In the human and rodent lung, the ECM is composed primarily of Col1 and Col3 subtypes, which provide the structural framework and elasticity of the alveolar wall (Amenta et al., 1988; Booth et al., 2012; Tsukui et al., 2020). In contrast to progressive increases in PSR staining and maintained overexpression of Col3, levels of Col1a1 and α‐SMA [marker for collagen‐producing cells of myofibroblast lineage (Wollin et al., 2015)] declined after reaching a maximum on day 14 in BLEO IPF mice. Global gene expression analysis confirmed temporal differences in *Col1* and *Col3* expression and further emphasized extensive alterations in ECM remodeling markers involving a wide range of collagen isoforms. As collagen fibers are composed of heterotypic fibrils and PSR binds to the fibrillary portion of various collagen types, including Col1 and Col3 (Junqueira et al., 1979), PSR staining may better capture collagen remodeling activity in BLEO‐IPF mice. Overall, spontaneous resolution of fibrosis and gradual recovery of pulmonary compliance in BLEO‐IPF mice is likely due to a combination of reduced fibrogenesis and increased collagen clearance.

In the present study, lung pathological analyses were applied to the right superior (biochemistry) and left (histology) lobule making it pertinent to validate if the changes observed were representative for the whole lung. We confirmed that changes in HP content and histological markers of fibrosis and inflammation were similar throughout the whole lung of BLEO‐IPF mice compared to normal controls, suggesting that BLEO distributed relatively equally to all parts of the lung upon intratracheal instillation. Using stereological sampling principles, we further verified that quantitative histological changes assessed by single section histomorphometry, as exemplified by Col1a1, were representative for the whole lung.

RNA sequencing data were also probed for an extensive set of genes linked to human lung fibrosis, including IPF (Ma et al., 2022; McDonough et al., 2019; Roach et al., 2021; Sivakumar et al., 2019; Wollin et al., 2015; Zhao et al., 2022). To a large extent, BLEO‐IPF mice and IPF patients showed similar directional shifts in the expression of these genes, lending further support to the use of the BLEO‐IPF mouse model in preclinical target discovery. Consistent with TGF‐β being a master regulator of fibrosis (Akhurst & Hata, 2012), BLEO‐IPF mice demonstrated substantially increased TGFβ levels in both BALF (TGFβ1, TGFβ2, TGFβ3) and lung tissue (TGFβ2, TGFβ3) for up to 28 days after BLEO administration. While *Tgfbr1* and *Tgfb2r* expression remained high, *Tgfbr3* was consistently downregulated in BLEO‐IPF mice. In comparison, the expression of *Smad* genes was largely unaffected (*Smad2‐3*) or downregulated (*Smad4‐7*). Smad proteins are canonical intracellular effectors of TGF‐β/TGFβR function but exhibit competing profibrotic and antifibrotic actions depending on the composition of TGFβR/Smad‐containing complexes. For example, TGFβ1R/Smad2‐3 interacts with Smad4 to promote myofibroblast activation (transcription of α‐SMA) and transcription of TGFβ1‐inducible ECM components, whereas Smad7 is negative regulator of Smad2/3 and inhibits fibrosis (Akhurst & Hata, 2012). In addition to the markedly elevated TGFβ protein levels clearly implicating TGFβRs in pulmonary fibrosis, our genome‐wide gene expression analysis signifies engagement of several TGFβ‐responsive genes in BLEO‐IPF mice. It should be noted that we did not assess TGFβR/Smad phosphorylation events which could serve to delineate TGFβ‐specific signaling events in the model further.

Rodent models applicable in preclinical drug discovery should demonstrate reproducible efficacy of a relevant drug treatment concept (Jenkins et al., 2017; Pound & Ritskes‐Hoitinga, 2018). Consequently, the repetition of intervention studies plays an integral role in the scrutinized validation of rodent models of IPF (Tashiro et al., 2017). As for clinical trials, measures to reduce group differences in baseline disease severity are often key to preventing bias and reducing variability in treatment outcomes in preclinical studies (Pound & Ritskes‐Hoitinga, 2018). Unrestrained WBP, a non‐invasive method allowing monitoring of respiratory mechanics in conscious mice (Hoymann, 2007; Lomask, 2006), has previously been reported to accurately detect respiratory deficits after single‐dose BLEO administration in mice (Khan et al., 2023; Milton et al., 2012; Vanoirbeek et al., 2010). Consistent with spirometry data, BLEO‐IPF mice also showed impaired respiratory function as measured by WBP. By detailed examination of WBP parameters, we confirmed that PenH is a sensitive proxy for impaired pulmonary function in BLEO‐IPF mice. As for spirometry variables, changes in WBP (PenH) regressed over time in BLEO‐IPF mice, although WBP‐assessed respiratory function remained significantly compromised for at least 28 days after single‐dose BLEO administration. Because gradual normalization of spirometry and WBP readouts occurred in parallel to spontaneous reversal of elevated lung weight, inflammation, and fibrosis, it may be speculated that both lung oedema and fibrosis were key determinants of spirometry and WBP outcomes in BLEO‐IPF mice. Importantly, increased PenH at baseline (day 7) was predictive of long‐term reductions in lung function in BLEO‐IPF mice, as measured by terminal spirometry on day 28. WBP (PenH) can therefore serve to exclude mice not responding adequately to BLEO as well as enable group‐wise stratification of lung disease before initiation of drug treatment in BLEO‐IPF mice. Baseline WBP (PenH)‐enabled group randomization and stratification was therefore applied in a comprehensive series of BLEO‐IPF mouse studies aiming to evaluate treatment efficacy of TGFβ1R‐selective blockade using an orally active TGFβ1R/ALK5i, (SB525334) (Grygielko et al., 2005).

ALK5i therapy was profiled using a moderate daily dose (60 mg/kg) in a 3‐week treatment intervention regimen (days 7–28 after the BLEO challenge), a time window applicable for studying the effects of lung fibrosis in the model. We initially confirmed the downregulation of pulmonary SMAD3 phosphorylation, a proxy for TGFβ1R target engagement, following ALK5i treatment in BLEO‐IPF mice. ALK5i also suppressed the expression of several TGFβ‐responsive genes, further supporting TGFβ‐directed inhibitory effects of the compound. ALK5i was therefore characterized in a total of 6 intervention studies with similar design for scrutinized assessment of the reproducibility of treatment outcomes following either twice‐daily (BID, 30 mg/kg, 3 studies) or once‐daily (QD, 60 mg/kg, 3 studies) administration over 3 weeks. Vehicle‐dosed BLEO‐IPF mice demonstrated comparable lung functional, biochemical, and histological changes across all intervention studies, further validating the robustness of the lung disease phenotype and enabling direct comparison of ALK5i effects in all intervention studies. Our study provides clear evidence of reproducible therapeutic efficacy of a standard TGFβR1/ALK5i in BLEO‐IPF mice, establishing ALK5i as a reliable reference drug in treatment intervention studies in the model. Irrespective of the dosing regimen applied, ALK5i consistently improved standard measures of lung function (FVC, FEV0.1, IC, static compliance) in BLEO‐IPF mice. FVC is considered most predictive for restrictive lung disease, and the most commonly employed and accepted primary endpoint in clinical trials for IPF (Fainberg et al., 2022; Nathan et al., 2021). In agreement with previous studies (Scotton et al., 2013), ALK5i did not reduce lung weight (oedema) in BLEO‐IPF mice.

Although there are no validated diagnostic or prognostic molecular markers for human interstitial lung diseases, biomarkers of ECM turnover/remodeling may hold promise in predicting lung function decline or outcome in patients with IPF (Inoue et al., 2020; Jenkins et al., 2015; Organ et al., 2019), emphasizing the focus on using more direct measures of lung fibrosis in clinical drug development. In contrast to clinical trials in IPF patients, lung biochemical and histological endpoints are commonly used in preclinical IPF studies, notably in various BLEO mouse models (Carrington et al., 2018; Della Latta et al., 2015). Among the extensive series of biochemical and histological markers applied to further validate the therapeutic effects of ALK5i, we report that two standard histological markers of fibrosis, i.e. total lung HP content and %‐area of PSR staining, were consistently improved. In agreement with our study, these markers have previously been reported reduced following ALK5i intervention therapy in BLEO mice (Jarman et al., 2014; Peng et al., 2013; Scotton et al., 2013; Smoktunowicz et al., 2015). Because total lung HP content and %‐area of PSR staining remained progressively elevated in the model, these two quantitative markers of fibrosis appear highly sensitive to detect antifibrotic drug effects. In contrast, the Ashcroft score and other quantitative histological markers (Col1a1, Col3, α‐SMA, Gal‐3) showed a spontaneous decline from day 14 after BLEO instillation (corresponding to ALK5i treatment day 7), which could render these markers less sensitive. Because reduced *Col1a1* and *Col3a1* mRNA expression has been reported following prophylactic (Cedilak et al., 2019; Higashiyama et al., 2007) and interventional (Jarman et al., 2014) ALK5i treatment in BLEO‐IPF mice, it may be speculated that ALK5i suppressed de novo collagen synthesis but was unable to enhance clearance of already deposited collagen fibers. Collectively, our in‐depth histological analyses indicates complex molecular mechanisms of ECM remodeling in BLEO‐IPF mice. Future histological studies must aim to characterize these dynamics in further detail.

In preclinical research, drug candidates for IPF have commonly been administered prior to BLEO instillation or within the early (inflammatory) phase of lung injury, that is, before the appearance of fibrotic lesions, thus aiming to prevent rather than reverse fibrosis (Kolb et al., 2020). Although this study design can provide preclinical proof of concept, it has limited applicability to human IPF as most patients have a significant fibrotic burden at the time of clinical presentation (Ley et al., 2011). While the BLEO‐IPF mouse is considered the best preclinical model available for drug efficacy testing (Jenkins et al., 2017), it should be noted that there exists no consensus on an optimal study design for profiling the antifibrotic efficacy of test drugs in standard animal models of IPF, including BLEO models. In the context of ALK5i treatment, previously reported BLEO studies vary substantially with regards to selection of rodent species (mouse or rat), BLEO challenge (intratracheal or intranasal; 6‐50 U per animal; 0.03–3 mg/kg) and ALK5i treatment regimen, including dose (10–60 mg/kg/day; oral or in‐diet), oral dosing frequency (BID or QD) and timing (prevention or intervention) and duration of treatment (7‐21 days) (Cedilak et al., 2019; Higashiyama et al., 2007; Jarman et al., 2014; Peng et al., 2013; Scotton et al., 2013; Smoktunowicz et al., 2015). Consistent with the common lack of pulmonary function tests in preclinical studies for IPF, only a single study has reported the use of spirometry to assess benefits of ALK5i treatment in BLEO‐IPF mice (Jarman et al., 2014). Our study, therefore, sets a framework for characterizing preclinical drug candidates for potential antifibrotic efficacy in the BLEO‐IPF mouse, using ALK5i as a reliable reference drug in treatment intervention studies.

The study has potential limitations. While we here provide a comprehensive description of the lung disease phenotype and reproducible ALK5i treatment responses in male BLEO‐IPF mice, future studies in BLEO‐IPF mice would benefit from comparatively profiling the model and ALK5i treatment in male vs. female mice. Given that aging is recognized as a major risk factor for IPF (Ley et al., 2011), translatability of the spirometry‐confirmed BLEO‐IPF mouse model may be further addressed by directly comparing the disease phenotype in young vs. aged mice (Redente et al., 2011). Considering that the single‐dose BLEO‐induced mouse model is limited by spontaneous resolution of lung disease, it could be advantageous to additionally profile ALK5i therapy in the context of sustained, progressive fibrotic lung injury, which has been reported following repetitive BLEO administration in mice (Redente et al., 2021). While we implemented non‐invasive WPB (PenH) with the aim to select and stratify BLEO‐IPF mice with impaired respiratory function at baseline, co‐application of WBP at study termination could potentially allow for within‐subject (pre‐to‐post) evaluation of lung function over the course of ALK5i treatment. Furthermore, similar scrutinized and repeated studies in the spirometry‐confirmed BLEO‐IPF mouse are warranted to inform about the degree of reproducibility of treatment outcomes using currently approved drugs for IPF, including pirfenidone and nintedanib.

## 5 CONCLUSION

The single intratracheal BLEO‐instillation mouse model of IPF consistently presents with impaired lung function and pulmonary fibrosis. ALK5i treatment reliably improves hallmarks of fibrotic lung disease, indicating the benefits of TGFβ signaling‐directed intervention in the model and establishing ALK5i as an applicable reference compound in BLEO‐IPF mouse studies. Furthermore, stratifying baseline WPB (PenH) readouts for confirmation of respiratory deficits has the potential to improve preclinical efficacy evaluation of novel drug candidates for IPF.

### AUTHOR CONTRUBUTIONS

AGP, SHK, MRR, HHH and MFE conceived and designed research. AGP, SHK, JB, DO, AMA, SEP, CGS, MWA, MRM, YN and JB performed experiments. AGP, SHK, JB, DO, AMA, SEP, CGS, MWA, MRM, YN, MRR and HHH analyzed data. AGP, SHK, AMA, CGS, MRM, MRR, US, HHH and MFE interpreted the results of the experiments. AGP, SHK, JB, DO, SEP, CGS, MWA, MRM, and HHH prepared figures. AGP, SHK, CGS, MWA, MRR, HHH and MFE drafted the manuscript. AGP, SHK, CGS, MWA, YN, MRR, US, HHH and MFE edited and revised the manuscript. All authors approved the final version of the manuscript.

## CONFLICT OF INTEREST STATEMENT

AGP, SHK, JB, DO, SEP, CGS, MWA, MRM, HHH and MFE are employed by Gubra; AGP, DO, CGS, MRM, HHH and MFE are shareholders in Gubra. AMA was employed by Gubra and is presently employed by IQVIA. YN, JB and MRR are employed by Enanta Pharmaceuticals; US is employed by Aarhus University, Aarhus, Denmark. No other potential conflicts of interest relevant to this article were reported.

## ETHICS STATEMENT

The Danish Animal Experimentation Council approved all experiments (licenses #2018‐15‐0201‐01532, #2023‐15‐0201‐01454). All animal experiments were conducted in accordance with Gubra bioethical guidelines which are fully compliant with national and internationally accepted principles for the ethical, humane and responsible care and use of laboratory animals. The animal facility at Gubra is a fully AAALAC accredited unit.

## Supporting information


Figure S1.
Figure S2.
Figure S3.
Figure S4.
Figure S5.
Figure S6.
Figure S7.
Figure S8.

Data S1.

## Data Availability

The RNA sequencing datasets generated in the current study are available in the Gene Expression Omnibus (GEO) repository (https://www.ncbi.nlm.nih.gov/geo/) with accession number GSE268757.
