# Supplementary material for: Reproducible lung protective effects of a TGFβR1/ALK5 inhibitor in a bleomycin‐induced and spirometry‐confirmed model of IPF in male mice
Source: Physiol Rep. 2024 Oct 11;12(19):e70077. doi: 10.14814/phy2.70077 (PMC11469938; doi:10.14814/phy2.70077)
Supplement: Supplementary file 1 — Figure S1. Figure S2. Figure S3. Figure S4. Figure S5. Figure S6. Figure S7. Figure S8. [file PHY2-12-e70077-s002.pdf]

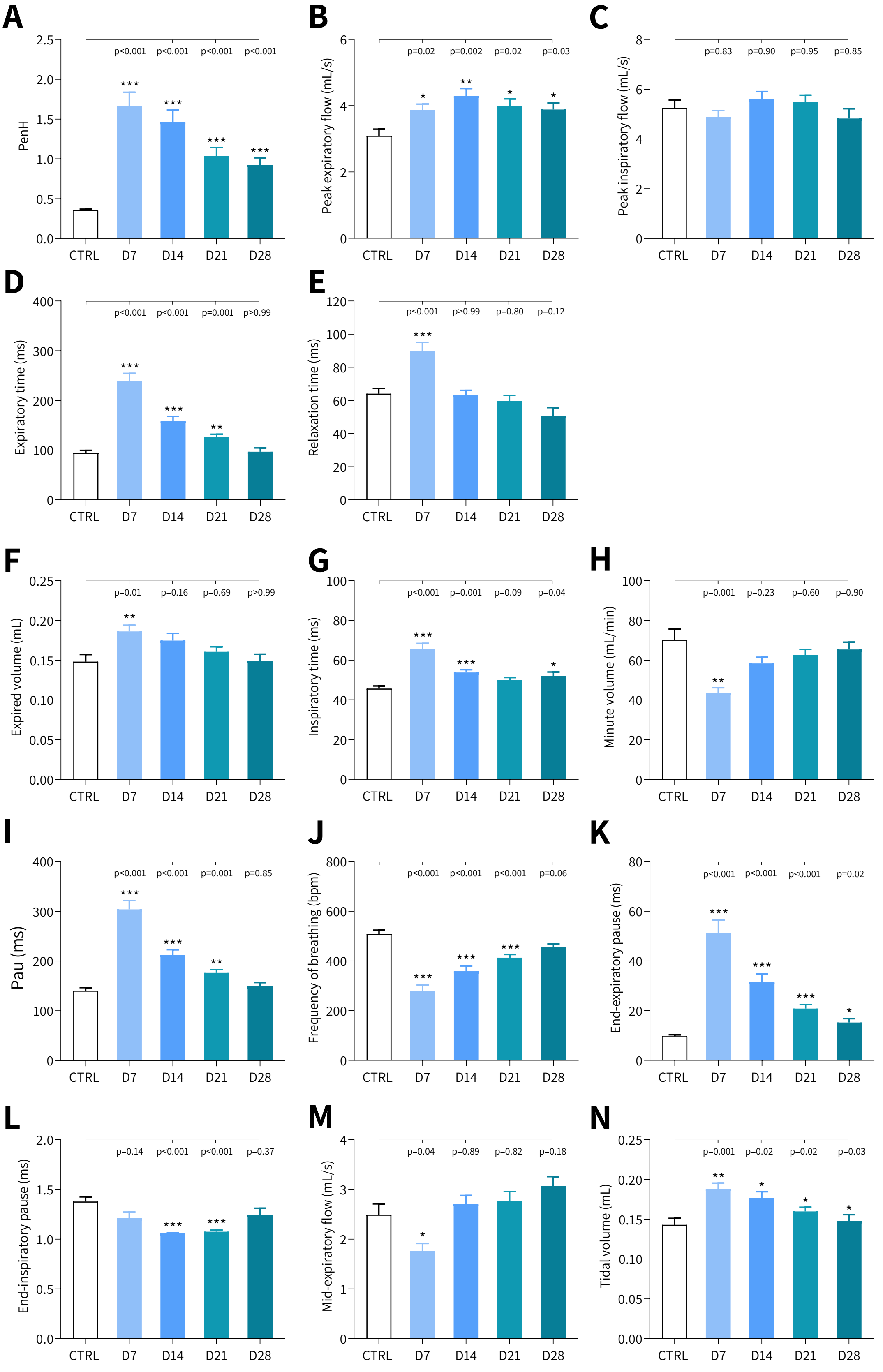

Figure S1. Application of non-invasive whole-body plethysmography for assessment of respiratory function in freely moving BLEO-IPF mice. Mice received an intratracheal installation of saline vehicle (CTRL, n=10) or bleomycin (2.0 mg/kg, n=11) on study day 1. Whole-body plethysmography (WBP) parameters were assessed on day 7-28 (D7-D28) after bleomycin administration. **(A)** PenH. **(B-E)** WBP parameters used for calculation of PenH. **(F-N)** Other parameters derived from the WBP analysis. Mean ± SEM. *p<0.05, **p<0.01, ***p<0.001 vs. CTRL. One-way ANOVA with Tukey's post-hoc test.

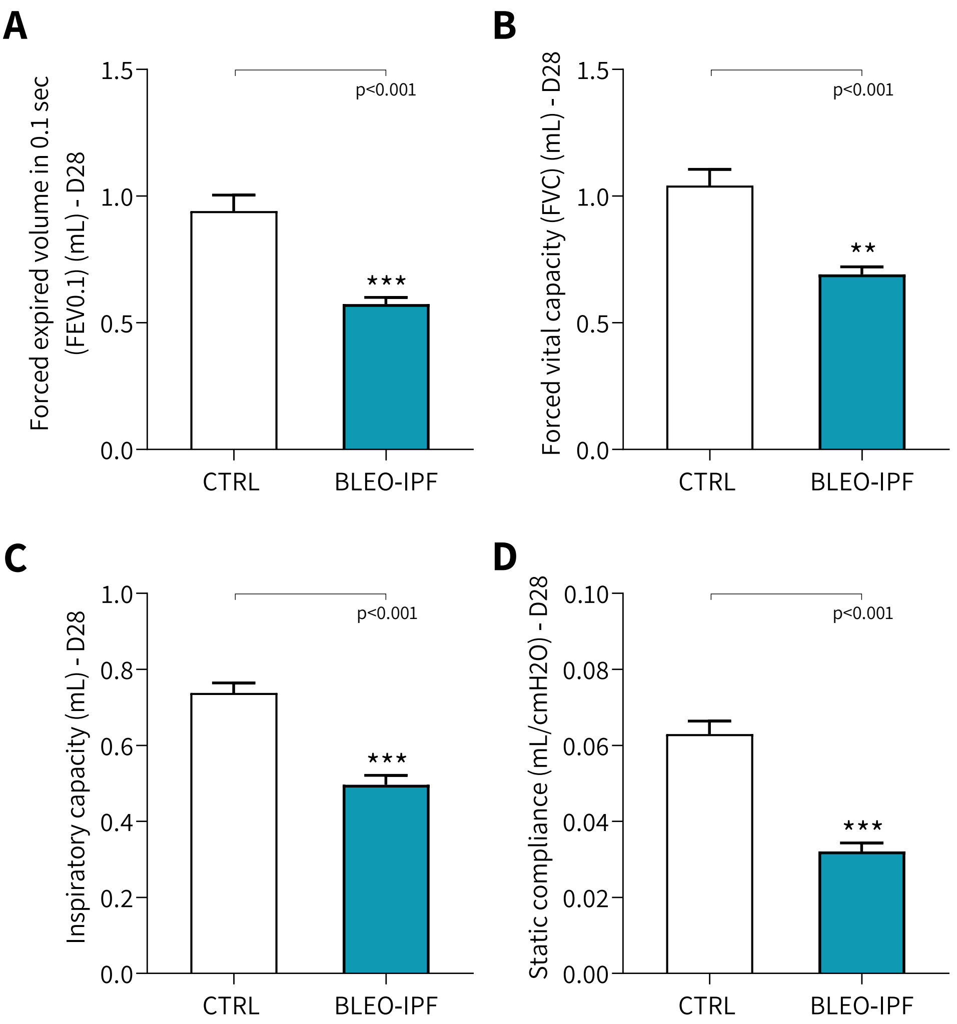

Figure S2. Spirometry performed at termination of longitudinal whole-body plethysmography study. Spirometry parameters were assessed on day 28 after an intratracheal installation of saline vehicle (CTRL, n=10) or bleomycin (BLEO-IPF, 2.0 mg/kg, n=11)**. (A)** Forced vital capacity (FVC). **(B)** Forced expiratory volume in 0.1 seconds (FEV0.1). **(E)** Inspiratory capacity. **(D)** Static compliance. (IC). Mean ± SEM. **p<0.01, ***p<0.001 vs. CTRL. Mann-Whitney test.

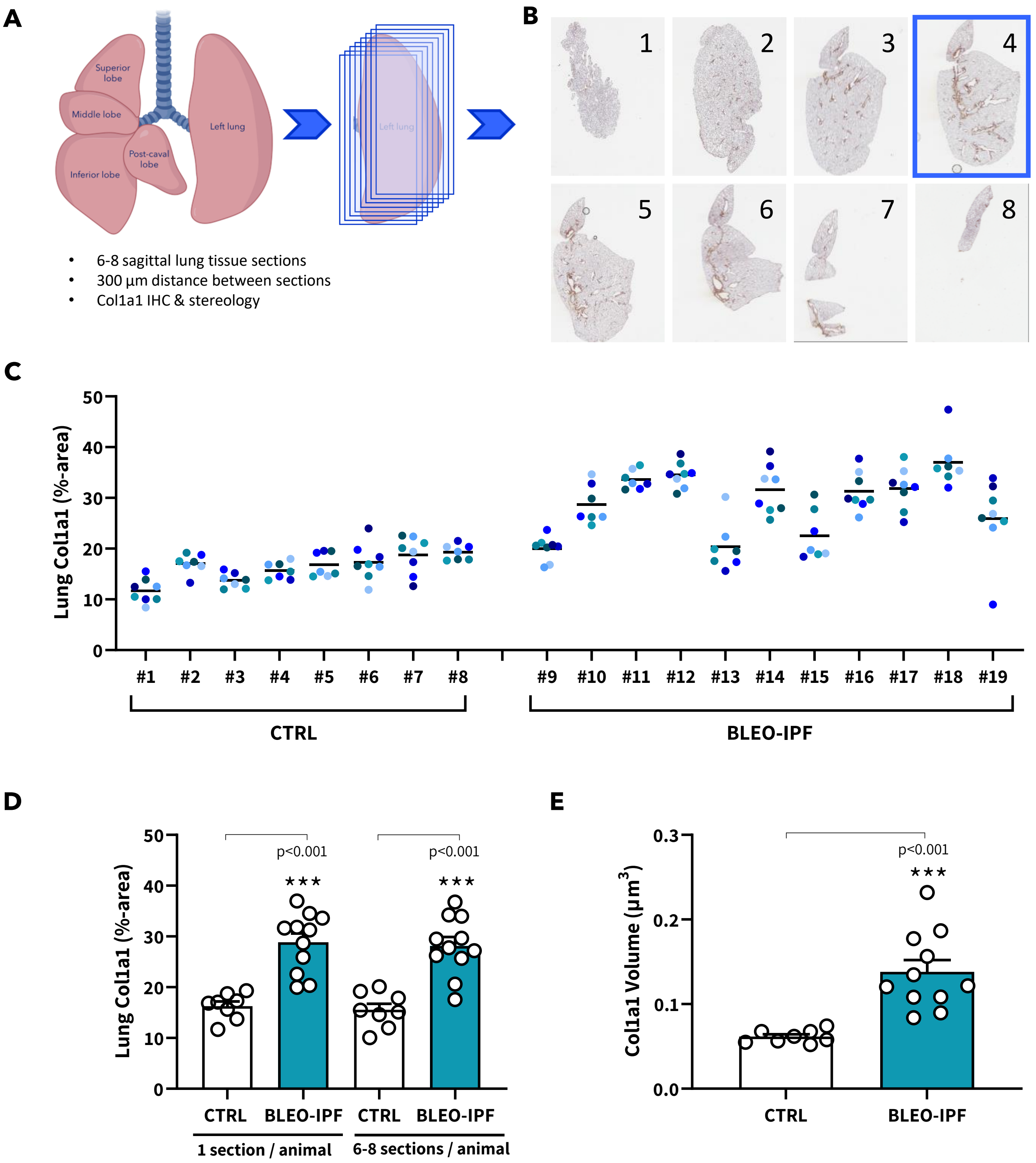

Figure S3. Collagen-1a1 histomorphometry on left lung single-sections is representative of whole-lobule collagen-1a1 expression levels in BLEO-IPF mice. Mice received an intratracheal installation of saline vehicle (CTRL, n=8) or bleomycin (BLEO-IPF, 2.0 mg/kg, n=11). Mice were terminated 28 days post-administration. **(A, B)** Using stereological principles, sagittal tissue sections (n=6-8 per animal) were systematically sampled throughout the left lung lobule and processed for collagen-1a1 (Col1a1) immunohistochemistry. **(C)** Proportionate (%) area of Col1a1 immunostaining at each lung sagittal level in individual CTRL and BLEO-IPF mice. **(D)** Comparison of Col1a1 %-area for representative lung section (1 section per animal) vs. mean of all sections sampled in each mouse. **(E)** Total lung lobule Col1a1 volume (μm^3 ± S.E.M.). ***$p<0.001$ vs. CTRL, one-way analysis of variance (ANOVA) for multiple comparisons followed by Tukey's post-hoc test. ns, not statistically significant ($p>0.05$).

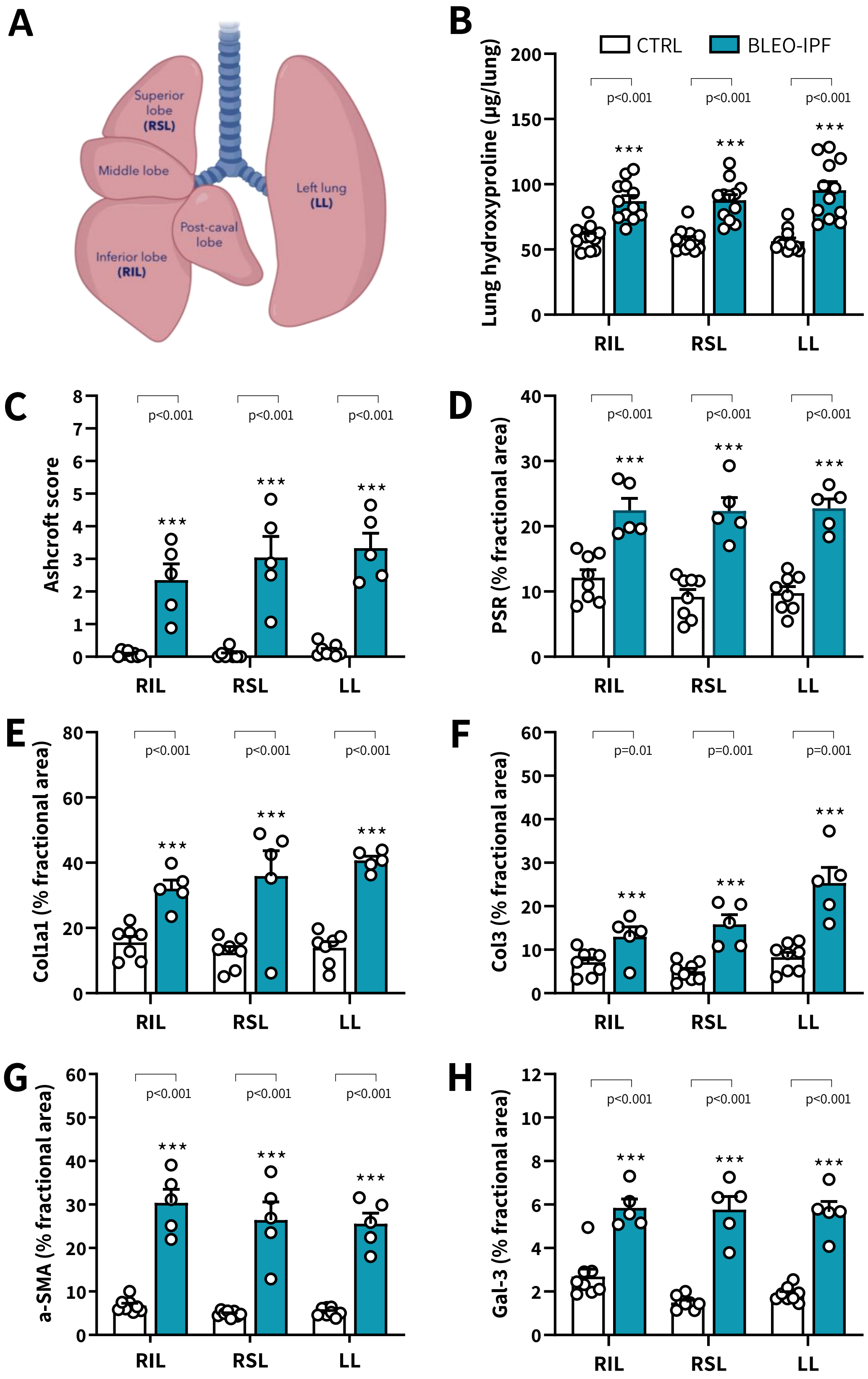

Figure S4. Assessment of left lobule histopathology is representative of whole-lung histopathology in BLEO-IPF mice. Mice received an intratracheal installation of saline vehicle (CTRL, n=6) or bleomycin (BLEO-IPF, 1.5 mg/kg, n=5) and terminated 28 days post-administration. **(A)** Sagittal tissue sections (n=1 per animal) were sampled from the right inferior (RIL), right superior (RSL) and left lung (LLL) lobule in CTRL and BLEO-IPF mice. **(B)** Hydroxyproline. **(C-H)** Sections were processed for Picro-sirius red (PSR) and Masson's trichrome (MT) staining as well as collagen-1a1 (Col1a1), collagen-3 (Col3), α-smooth muscle actin (α-SMA) and galectin-3 (Gal-3) immunohistochemistry. **(C)** Ashcroft score (analysed from MT-stained sections). Proportionate (%) area of **(D)** PSR staining, **(E)** Col1a1, **(F)** Col3, **(G)** α-SMA, and **(H)** galectin-3. Mean ± SEM. *p<0.05, **p<0.01, ***p<0.001 vs. CTRL, one-way analysis of variance (ANOVA) for multiple comparisons followed by Tukey's post-hoc test. ns, not statistically significant (p>0.05).

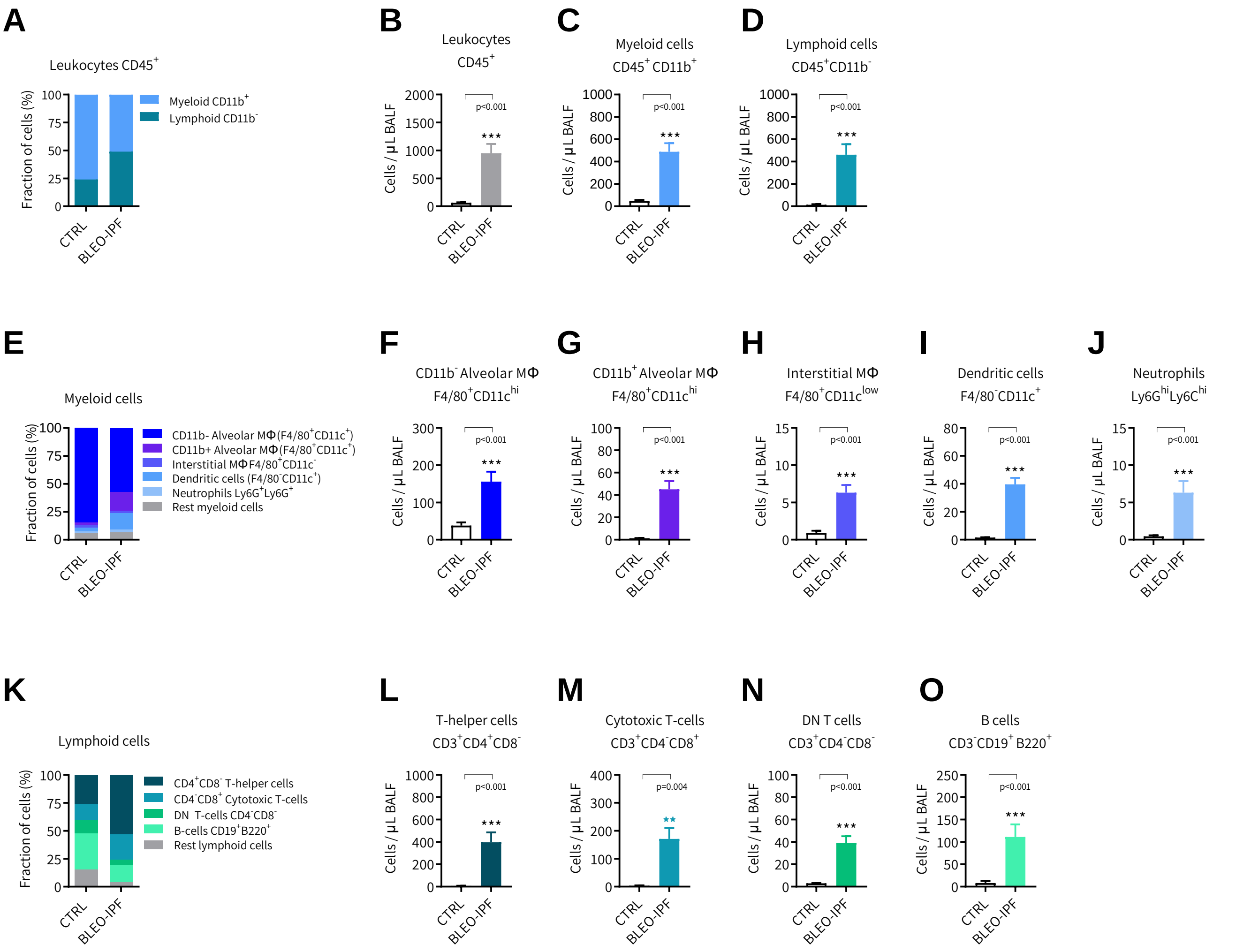

Figure S5. BLEO-IPF mice show marked expansions in BALF-derived macrophage and lymphoid subsets. Mice received an intratracheal installation of saline vehicle (CTRL, n=10) or bleomycin (BLEO-IPF, 1.5 mg/kg, n=13) and were terminated 21 days post-administration. **(A-D)** Relative proportions (%, cells/µL BALF) of leukocytes, myeloid and lymphoid cells. **(E-J)** Distribution (%, cells/µL BALF) of myeloid cell types, including tissue resident (alveolar)- and infiltrating (interstitial) monocytes/macrophages, dendritic-like cells and neutrophils. **(K-O)** Distribution (%, cells/µL BALF) of lymphoid cell types, including T-helper cells, cytotoxic T cells, double-negative (DN) T cells and B cells. **p<0.01, ***p<0.001 vs. CTRL (Dunnett's test one-factor linear model).

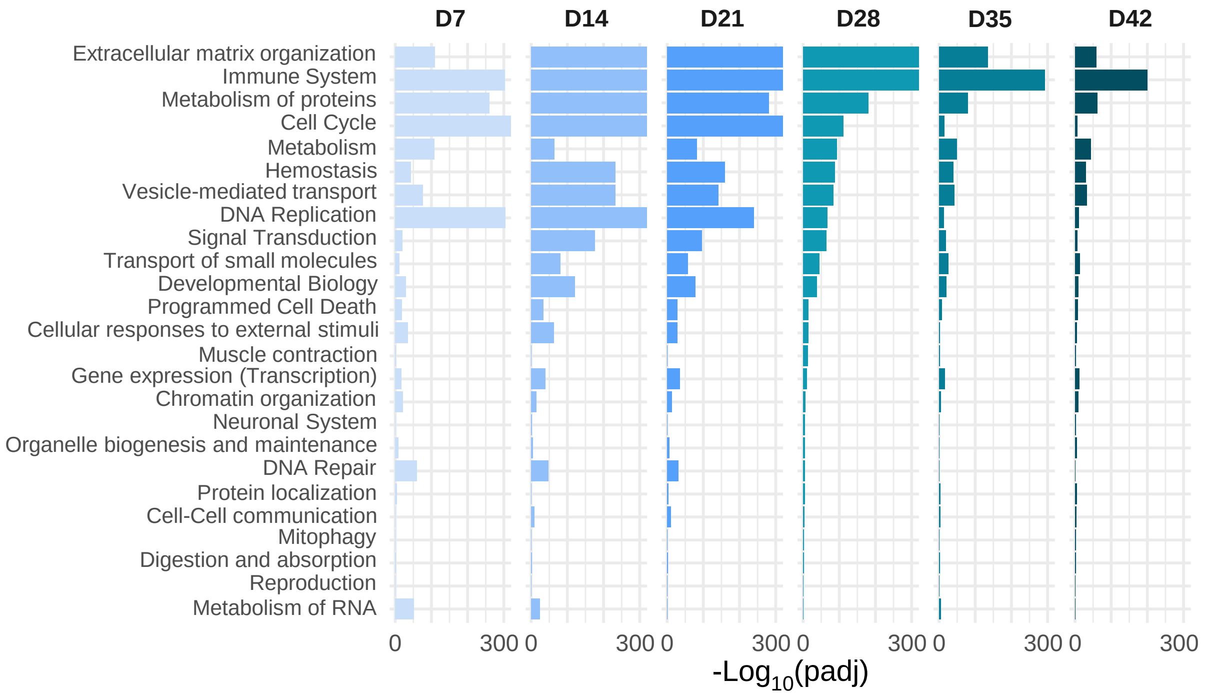

Figure S6. Progressive lung transcriptome changes in BLEO-IPF mice. Top-level Reactome pathway enrichment analysis of progressive lung transcriptome changes in BLEO-IPF mice as compared to control mice (intratracheal saline administration). Pathways are ranked according to perturbations on day 28 (D28). See Fig. S7 for further resolution of pathway enrichment within Extracellular matrix organisation and Immune System.

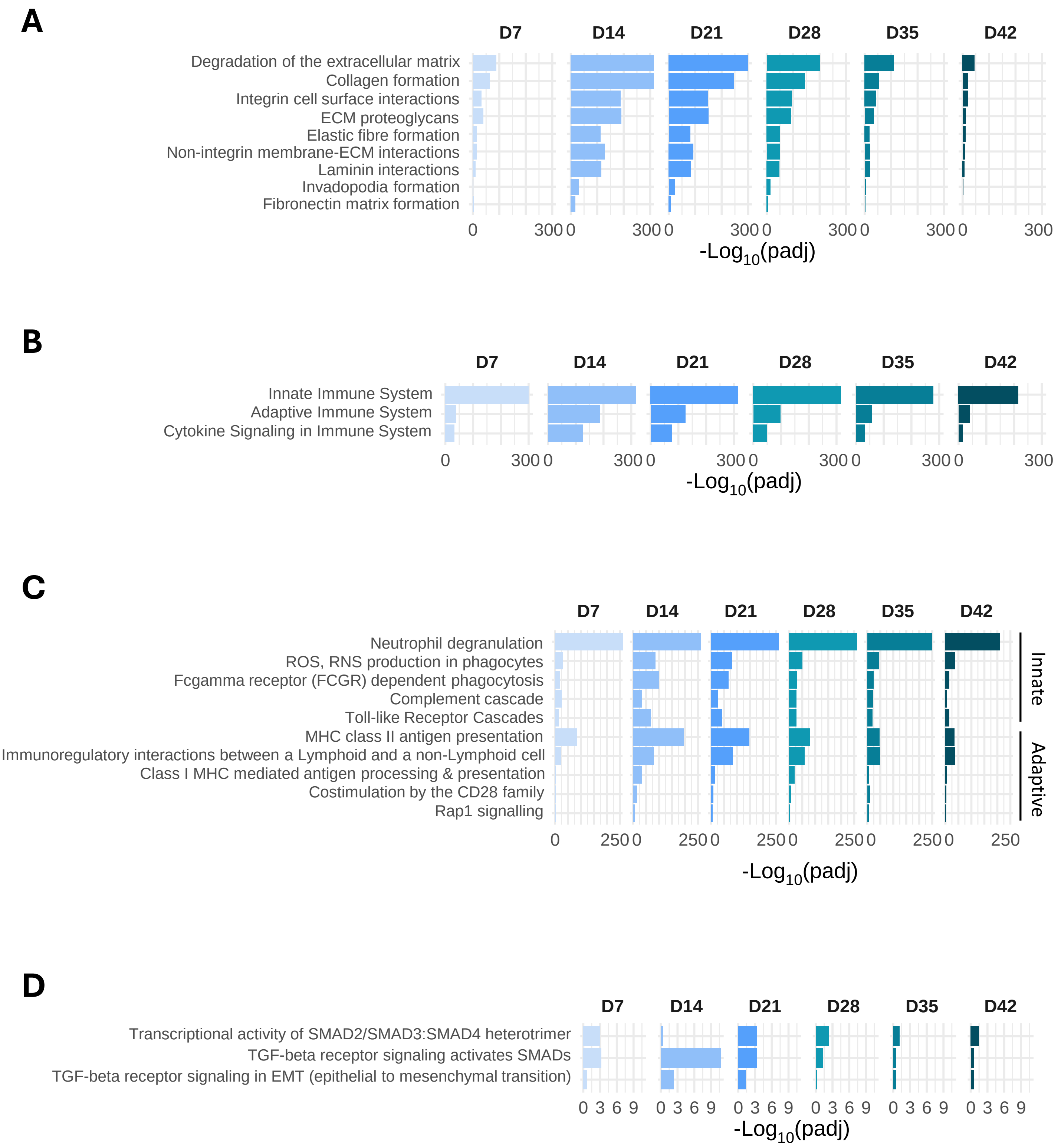

Figure S7. Progressive lung transcriptome changes in BLEO-IPF mice. Progressive enrichment of extracellular matrix (ECM) organisation and immune system pathways in BLEO-IPF mice as compared to control mice (intratracheal saline administration). Pathways are ranked [-$\log_{10}$ (adjusted p-value)] according to perturbations on day 28 (D28). **(A)** ECM organisation. **(B)** Top-level immune system pathways. **(C)** Top-5 pathway enrichment for the innate and adapative immune system, respectively. **(D)** Signaling by TGF-beta Receptor Complex subpathways.

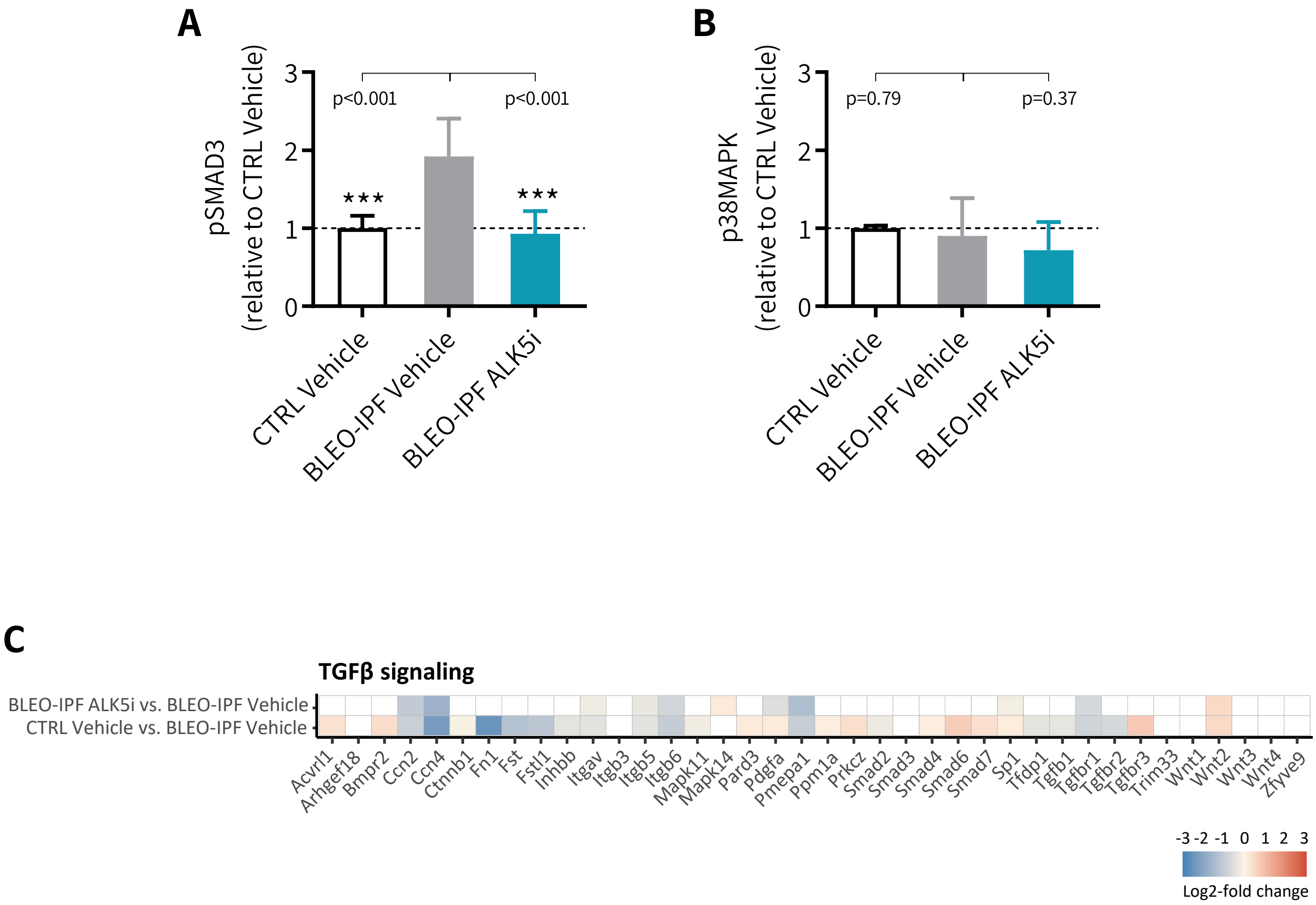

Figure S8. Pharmacodynamic biomarkers of pulmonary TGFβR1/ALK5 target engagement assessed in BLEO-IPF mice with spirometry-confirmed lung injury. Mice received bi-daily (BID) oral administration (5 ml/kg) of vehicle (n=14) or ALK5i (30 mg/kg, n=13) for 21 days, starting on day 7 after an intratracheal installation of bleomycin (BLEO-IPF, 2.0 mg/kg). Mice administered vehicle (BID) served as controls (CTRL Vehicle, n=10). **(A, B)** SMAD3 and p38MAPK phosphorylation levels expressed relative to CTRL Vehicle. Lung p-SMAD3 and p-38MAPK levels were analyzed using a one-way analysis of variance (ANOVA) followed by Dunnett's multiple comparisons test. Data are indicated a mean ± S.E.M. ***$p<0.001$ vs. BLEO-IPF Vehicle. **(C)** Gene expression markers of TGFβ signaling assessed by RNA sequencing and expressed as RPKM (reads per kilobase per million mapped reads). Lung gene expression data were analyzed using the R package DESeq2 with p-values corrected for multiple testing using the Benjamini-Hochberg method (5% false discovery rate). Color gradients indicate significantly upregulated (red color) or downregulated (blue color) genes in BLEO-IPF mice compared to BLEO-IPF Vehicle control mice. White color indicates no significant change in gene expression compared to BLEO-IPF Vehicle control mice.
