# Supplementary material for: Reproducible lung protective effects of a TGFβR1/ALK5 inhibitor in a bleomycin‐induced and spirometry‐confirmed model of IPF in male mice
Source: Physiol Rep. 2024 Oct 11;12(19):e70077. doi: 10.14814/phy2.70077 (PMC11469938; doi:10.14814/phy2.70077)
Supplement: Supplementary file 2 — Data S1. [file PHY2-12-e70077-s001.docx]

| **Parameter** | **Feature** | **BLEO-IPF mice** | **References** |
| --- | --- | --- | --- |
|  | **Spirometry** |  |  |
| *FVC (mL)* | Forced vital capacity (FVC) is the total volume expired during a forced expiration. | ↓ | 1-4 |
| *FEV (mL)* | Forced Expired Volume (FEV0.1) is the volume expired during the first 0.1 seconds of a forced expiration. | ↓ |  |
| *IC (mL)* | Inspiratory Capacity (IC) is the amount of air that can be inhaled after the end of a normal expiration. | ↓ |  |
| *Cst (mL/cmH2O)* | Static compliance (Cst) is a parameter extracted from a pressure-volume (PV) curve. Measured under closed-chest conditions, it reflects the intrinsic elastic properties of the respiratory system (i.e. lung + chest wall) at rest. | ↓ |  |
| *Flow-volume (FV) curve* | Generated by inflating the animal’s lungs to a pressure of 30 cm H2O over 1 s and held at that pressure for 0.5 s. The animal’s lungs are then rapidly exposed to a negative pressure for another 0.5 s (while volume, flow and pressure changes are recorded). A FV curve is generated, and parameters such as FVC and FEV0.1 are calculated. | ↑ |  |
| *Pressure-volume curves/loops* | During a ramp-style PV curve, the subject’s lungs are inflated and deflated at a constant and steady flow rate. PV curves assess the distensibility of the respiratory system at rest over the entire inspiratory capacity (i.e. from end of expiration to total lung capacity). | ↓ |  |
|  | **Whole-body plethysmography (WBP)** |  |  |
| *PenH* | Enhanced pause (PenH) is a unit-less and dimensionless parameter that combines both time and flow rates to describe the shape of box flow and thus ventilation. Pause is defined as ((Te-RT)/RT) and enhanced pause (Penh) is pause multiplied by (PEF/PIF). Penh is influenced by changes occurring in both nasal cavity and thoracic cavity | ↑ | 5-6 |
| *PIF (mL)* | Peak inspiratory flow (PIF) is the largest measured flow during the inspiratory portion of the breath | ↔ |  |
| *PEF (mL)* | Peak expiratory flow (PEF) is the largest measured flow during the expiratory portion of the breath | ↑ |  |
| *RT (sec)* | Relaxation time (RT) is the time required to exhale 74% of the tidal volume | ↑ |  |
| *Te (sec)* | Expiratory time (Te) is the amount of time required for the animal to exhale (time from end of inspiration to start of next inspiration). | ↑ |  |
| *EV (mL)* | Expired volume (EV) is the calculated expiratory volume (area under the flow curve). | ↑ |  |
| *Ti (ms)* | Inspiratory time (Ti) is the amount of time required for the animal to inhale. | ↑ |  |
| *Minute volume (mL/min)* | Minute volume is the total volume of air inhaled or exhaled in one minute. | ↓ |  |
| *Pau (ms)* | Pause (Pau) is defined as ((Te-RT)/RT). | ↑ |  |
| *Frequency of breathing (bpm)* | Breathing frequency is measured as the inverse of the sum of time of  inspiration and expiration. Respiratory Rate is the number of breaths taken by the subject per minute, the units are breaths per minute (bpm). | ↓ |  |
| *EEP (ms)* | End-expiratory pause (EEP) is the amount of time at no flow following expiration. | ↑ |  |
| *EIP (ms)* | End-inspiratory pause (EIP) is the amount of time at no flow following inspiration. | ↓ |  |
| *MEF (mL/s)* | Mid-expiratory flow (MEF) is the rate of airflow during the middle one half of the expiratory portion of the breath (between 25% and 75% of the volume expired). | ↓ |  |
| *TV (mL)* | Tidal volume (TV) is the calculated inspiratory volume (area under the flow curve). | ↑ |  |

**Table S1.** Definition and interpretation of spirometry and whole-body plethysmography variables applied in the study. Symbols: ↑ (increased); ↓ (reduced); ↔ (no change). References are listed below. See Fig. 2 (spirometry) and Fig. S1 (whole-body plethysmography) for the corresponding longitudinal lung functional data in BLEO-IPF mice.

**References**

1. West JB. (2012) Chapter 7: Mechanics of Breathing—How the Lung Is Supported and Moved. In *Respiratory Physiology: The Essentials*, 9th ed. Philadelphia: Wolters Kluwer Health/Lippincott Williams and Wilkins.

1. Reiss LK, Kowalik A, Uhlig S. (2011) Recurrent recruitment manoeuvres improve lung mechanics and minimize lung injury during mechanical ventilation of healthy mice. *PLoS One* 6: e24527.
2. Vanoirbeek YF et al. (2010) Noninvasive and invasive pulmonary function in mouse models of obstructive and restrictive respiratory diseases. *Am J Respir Cell Mol Biol* 42: 96-104.
3. Devos FC et al. (2017) Forced expiration measurements in mouse models of obstructive and restrictive lung diseases. *Respir Res* 18: 123.
4. Mitzner W, Tankersley C. (2003) Interpreting Penh in mice. *J Appl Physiol* 94:828-832.
5. Bates JH, Irvin CG. (2003) Measuring lung function in mice: the phenotyping uncertainty principle. *J Appl Physiol* 94(4):1297–1306.

| **Fibrosis grade** | **Histological features** |
| --- | --- |
| **0** | Normal lung tissue |
| **1** | Minimal fibrotic changes (alveolar septa ≤3× thicker than normal) |
| **2** | Clearly fibrotic changes (alveolar septa >3× thicker than normal) with knot-like formation but not connected to each other |
| **3** | Contiguous fibrotic walls (alveolar septa >3× thicker than normal) predominantly in whole microscopic field |
| **4** | Single fibrotic masses (≤10% of microscopic field) |
| **5** | Confluent fibrotic masses (>10% and ≤50% of microscopic field) |
| **6** | Large contiguous fibrotic masses (>50% of microscopic field). Lung architecture mostly not preserved |
| **7** | Non-existent alveolar septa. Alveoli nearly obliterated with fibrous masses but still up to five air bubbles |
| **8** | Microscopic field with complete obliteration with fibrotic masses |

**Table S2.** Criteria for grading lung fibrosis as outlined by Ashcroft *et al.* (J Clin Pathol 41: 467–470, 1988) and Hübner *et al.* (Biotechniques 44: 507-511, 2008).
